# Identification of macrophages in normal and injured mouse tissues using reporter lines and antibodies

**DOI:** 10.1038/s41598-022-08278-x

**Published:** 2022-03-16

**Authors:** Bijun Chen, Ruoshui Li, Akihiko Kubota, Linda Alex, Nikolaos G. Frangogiannis

**Affiliations:** The Wilf Family Cardiovascular Research Institute, Department of Medicine (Cardiology), Albert Einstein College of Medicine, 1300 Morris Park Avenue Forchheimer G46B, Bronx, NY 10461 USA

**Keywords:** Immunology, Cardiology, Diseases

## Abstract

Reliable tools for macrophage identification in mouse tissues are critical for studies investigating inflammatory and reparative responses. Transgenic reporter mice and anti-macrophage antibodies have been used as “specific pan-macrophage” markers in many studies; however, organ-specific patterns of expression and non-specific labeling of other cell types, such as fibroblasts, may limit their usefulness. Our study provides a systematic comparison of macrophage labeling patterns in normal and injured mouse tissues, using the CX3CR1 and CSF1R macrophage reporter lines and anti-macrophage antibodies. Moreover, we tested the specificity of macrophage antibodies using the fibroblast-specific PDGFR$\mathrm{\alpha }$ reporter line. Mouse macrophages exhibit organ-specific differences in expression of macrophage markers. Hepatic macrophages are labeled for CSF1R, Mac2 and F4/80, but lack CX3CR1 expression, whereas in the lung, the CSF1R+/Mac2+/Mac3+ macrophage population is not labeled with F4/80. In the splenic red pulp, subpopulations of CSF1R+/F4/80+/Mac3+cells were labeled with Mac2, CX3CR1 and lysozyme M. In the kidney, Mac2, Mac3 and lysozyme M labeled a fraction of the CSF1R+ and CX3CR1+ macrophages, but also stained tubular epithelial cells. In normal hearts, the majority of CSF1R+ and CX3CR1+ cells were not detected with anti-macrophage antibodies. Myocardial infarction was associated with marked expansion of the CSF1R+ and CX3CR1+ populations that peaked during the proliferative phase of cardiac repair, and also expressed Mac2, Mac3 and lysozyme M. In normal mouse tissues, a small fraction of cells labeled with anti-macrophage antibodies were identified as PDGFR$\mathrm{\alpha }$+ fibroblasts, using a reporter system. The population of PDGFR$\mathrm{\alpha }$+ cells expressing macrophage markers expanded following injury, likely reflecting emergence of cellular phenotypes with both fibroblast and macrophage characteristics. In conclusion, mouse macrophages exhibit remarkable heterogeneity. Selection of the most appropriate markers for identification of macrophages in mouse tissues is dependent on the organ and the pathologic condition studied.

## Introduction

Macrophages are specialized phagocytes that recognize, engulf and digest microbes, dying cells and cellular debris, cancer cells and foreign bodies, thus clearing tissues from damaged cells and microorganisms^1^. In addition to their role in phagocytosis, macrophages are also key cellular effectors in both innate and adaptive immunity, initiating and regulating inflammatory reactions following injury, and mediating lymphocyte-dependent immune responses. In mammals, most tissues harbor significant populations of macrophages^2–4^. Abundant macrophages are strategically located in tissues involved in clearance of dead cells, foreign bodies or microbes, such as the spleen, lung, liver and gut and regulate responses to injury^5^. In addition, it has been suggested that in specialized organs, such as the heart, macrophage populations may play important homeostatic roles, facilitating conduction of the electrical impulse^6^ and preserving function^7^.

Dissection of the role of macrophages in homeostasis and disease requires reliable, specific and well-characterized animal models for their identification, fate mapping and cell-specific gene targeting. Several systems have been used to label and track macrophages in tissue sections, in order to derive conclusions regarding the role and fate of macrophages in homeostasis and disease. Differentiation, proliferation and survival of macrophages is controlled by an interaction between the cytokine Colony stimulating factor (CSF)1 and its receptor CSF1R^8^. CSF1R expression is low in hematopoietic stem cells^9^, increases in macrophage progenitors (colony forming unit macrophages – CFU-M), and then further increases gradually as CFU-M differentiate to monocytes and macrophages^8,10–12^. CSF1R reporter systems have been extensively used to label macrophages in tissues of mice^13–16^ rats^17^, chicken^18^ and sheep^19^. In mice, the *Csf1r* promoter region including the conserved Fms intronic regulatory element (FIRE) has been used to drive reporter transgenes^13,14^, inducible Cre recombinase^20^, or Fas-induced apoptosis^21^. The fractalkine receptor CX3CR1 has also been used as a marker of monocytes and macrophages. A CX3CR1 reporter mouse, in which the *Cx3cr1* gene has been replaced by GFP^22^ has been extensively used to label monocytes and macrophages. Lysozyme-M reporter mice and Cre recombinase drivers have been extensively used to trace myeloid cells and for macrophage-specific gene targeting^23–25^. Furthermore, several “macrophage-specific” antibodies, such as anti-Mac2 (Galectin-3), Mac3 (LAMP2/CD107b), F4/80 and anti-CD68 antibodies are routinely used to label macrophages in various mouse tissues^26^.

The reliability and specificity of these tools in detection of antibodies is debated. Although in many studies these tools are used as specific pan-macrophage markers^27–30^, other investigations have challenged their specificity and sensitivity for macrophage populations in various organs. For example, *Csf1r* mRNA (but not protein) expression by neutrophilic granulocytes^31^ limits the specificity of CSF1R reporter animals in macrophage labeling. Although CX3CR1 has been suggested to be a pan-macrophage marker in some studies^3,28^, the embryonically-derived^32^ macrophage populations in the liver and the peritoneum lack CX3CR1 expression. Moreover, some studies have suggested that several murine anti-macrophage antibodies (including Mac2, Mac3 and anti-CD68) may also detect other cell types (such as fibroblasts)^33^. These conflicting findings on the specificity and sensitivity of macrophage markers have generated confusion regarding the content of various tissues in macrophages, and the role of these cells in pathologic responses. For example, use of similar macrophage reporter models identified “abundant”^34^ cardiac macrophages in one study, but only “sparse” myocardial macrophages in another investigation^35^.

In the current study, we compared the specificity and reliability of CSF1R and CX3CR1 macrophage reporter models, and several macrophage-specific antibodies in labeling macrophages in health and disease. In order to examine the previously suggested cross-reactivity of macrophage markers with fibroblasts, we used the well-documented and specific PDGFR$\mathrm{\alpha }$-EGFP fibroblast reporter line^36,37^. Our findings suggest that macrophages exhibit organ-specific characteristics. Although macrophage markers show limited cross-reactivity with fibroblasts in normal tissues, a population of cells expressing both fibroblast and macrophage markers emerges following injury. Thus, there is no single optimally specific and reliable pan-macrophage marker. Design of studies to label, track and target macrophages requires understanding of their tissue-specific properties, and consideration of phenotypic changes occurring upon their activation.

## Results

### Identification of macrophages in normal mouse tissues using CSF1R-EGFP and CX3CR1^GFP^ reporter mice

**Figure 1:**
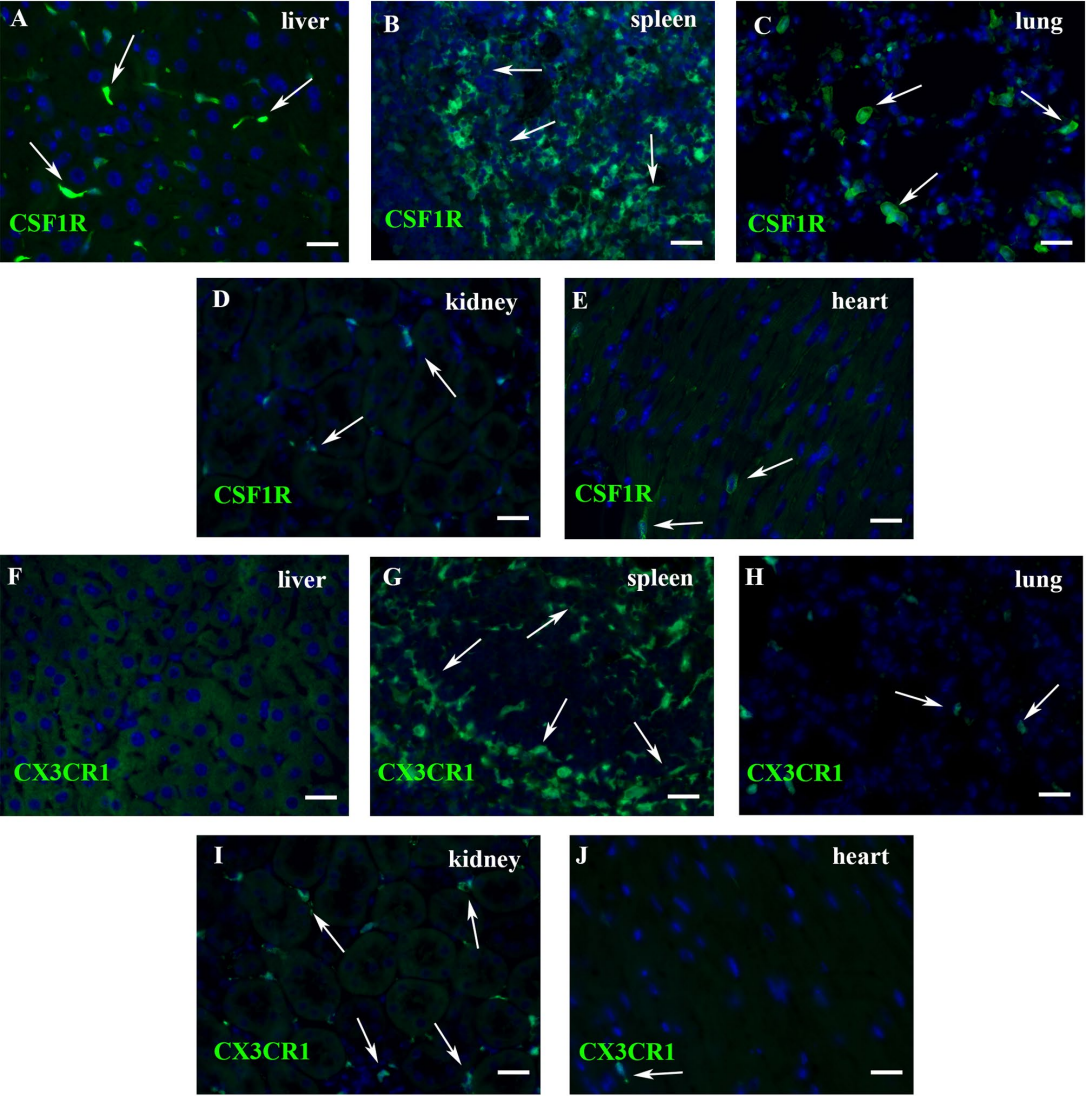
Use of the CSF1R-EGFP and CX3CR1^GFP^ reporter mice to identify mouse macrophages in normal tissues. A-E: Representative images show identification of CSF1R+ cells (arrows) in the liver (**A**), splenic red pulp (**B**), pulmonary parenchyma (**C**), kidney (**D**) and heart (**E**) of young adult CSF1R-EGFP mice. (**F**–**J**) Immunofluorescent staining of sections from CX3CR1^GFP^ reporter mice. Hepatic macrophages are not labeled for CX3CR1 (**F**). CX3CR1+ cells are identified in the white pulp of the spleen (**G**) and in the lung (**H**). Sparse populations of CX3CR1+ cells are found in the kidney (**I**) and in the heart (**J**). Scale bar: 20 μm.

We used anti-GFP staining in reporter mice to identify CSF1R+ (Fig. 1A–E) and CX3CR1+ (Fig. 1F–J) macrophages in adult mouse tissues. Abundant CSF1R+ cells were identified in the liver (Fig. 1A), splenic red pulp (Fig. 1B), and lung (Fig. 1C); much smaller populations were found in the kidney (Fig. 1D) and in the myocardium (Fig. 1E). No CX3CR1+ cells were found in the liver (Fig. 1F). Abundant CX3CR1+ cells were noted in the spleen and were localized mostly in the white pulp (Fig. 1G), with smaller populations in the lung (Fig. 1H), kidney (Fig. 1I) and myocardium (Fig. 1J).

### Hepatic macrophages express CSF1R, but not CX3CR1, and are optimally labeled with Mac2, F4/80 and anti-lysozyme antibodies

**Figure 2:**
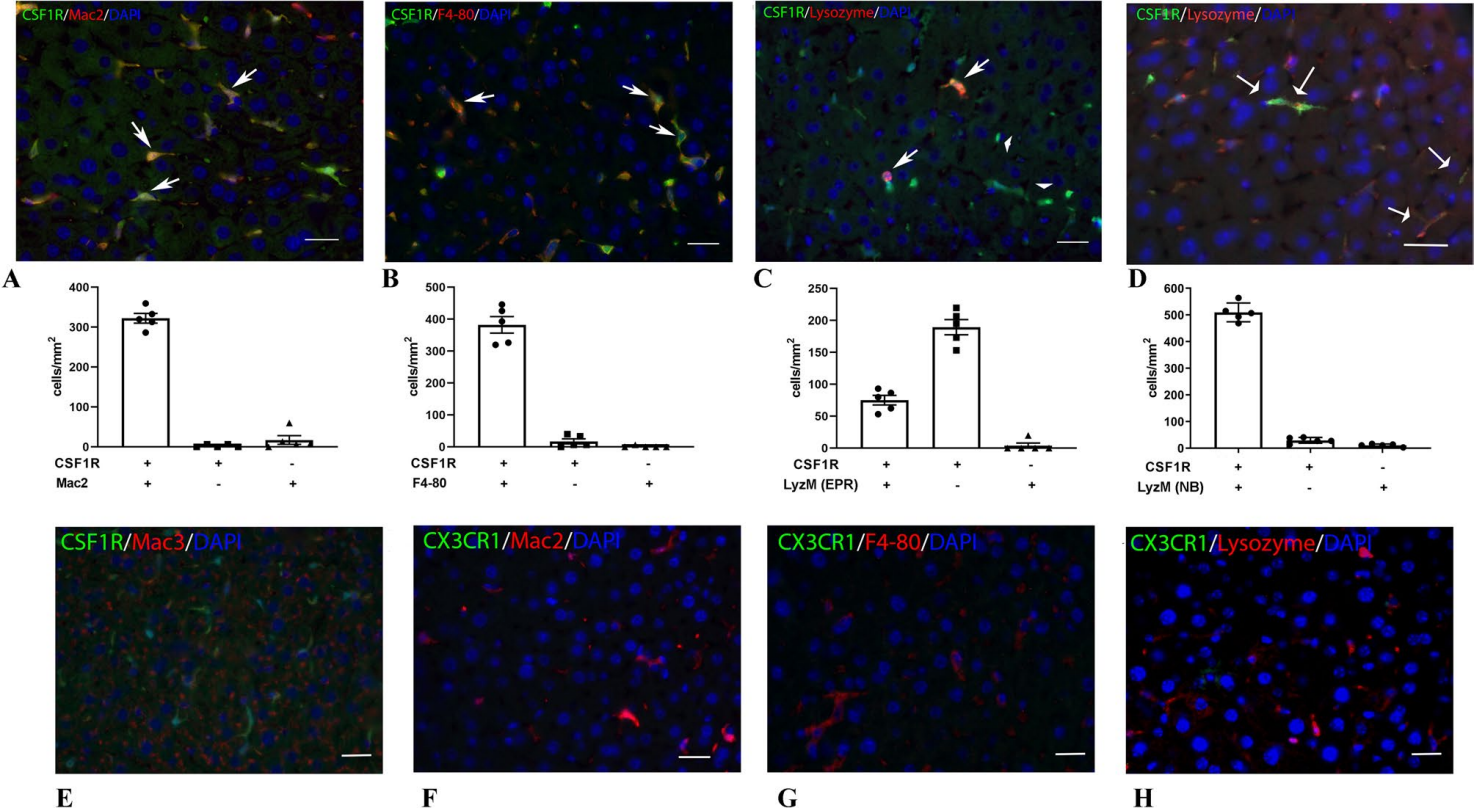
Identification of liver macrophages using antibodies and reporter lines. (**A**–**C**) Dual labeling for GFP and antibodies to Mac2 (**A**), F4/80 (**B**) and lysozyme M (**C**,**D**). Arrows show double positive cells. Quantitative analysis shows that Mac2 (**A**) and F4/80 (**B**) co-localize with CSF1R+ cells. In contrast, the majority of CSF1R+ cells are not labeled with the rabbit monoclonal anti-lysozyme antibody EPR2994(2) (EPR, C arrowheads). In order to examine whether the lack of lysozyme immunoreactivity in the majority of hepatic macrophages reflects the low sensitivity of the antibody, we also tested the rabbit polyclonal anti-lysozyme antibody NBP2-61,118 (NB, D). Quantitative analysis showed that virtually all hepatic macrophages exhibited lysozyme immunoreactivity for the NBP2-61,118 antibody. (**E**) A representative image shows that the antibody to Mac3 is not suitable for identification of hepatic macrophages, exhibiting punctate staining throughout the liver. (**F**–**H**) CX3CR1/Mac2, CX3CR1/F4-80 and CX3CR1/Lysozyme staining confirm the absence of CX3CR1 in hepatic macrophages. Scale bar: 20 μm.

Because liver macrophages do not express CX3CR1 (Fig. 1F), we used only the CSF1R-EGFP mice to characterize hepatic macrophages. Dual fluorescence studies showed that the virtually all CSF1R+ macrophages were labeled with the Mac2 (Fig. 2A) and F4/80 antibodies (Fig. 2B). In contrast, only 28.3% ± 2.3 of CSF1R+ cells stained with the rabbit monoclonal anti-lysozyme antibody EPR2994(2) (Fig. 2C). Because absence of lysozyme staining in liver macrophages could reflect low sensitivity of the antibody, we tested a second antibody against lysozyme in liver sections. Dual labeling using the rabbit polyclonal anti-lysozyme antibody NBP2-61,118 showed that virtually all CSF1R+ cells in the liver parenchyma are LyzM+ (Fig. 2D). Virtually all Mac2+, F4/80+ and LyzM+ cells were also labeled for CSF1R, suggesting that all 3 antibodies are specific for hepatic macrophages. Diffuse punctate Mac3 staining was noted throughout the liver (Fig. 2E), making it an unsuitable marker for identification of hepatic macrophages. Dual fluorescence in CX3CR1^EGFP^ mice confirmed the absence of CX3CR1 expression in Mac2+, F4/80+ and LyzM+ macrophages (Fig. 2F–H). In summary, hepatic macrophages express CSF1R, Mac2, F4/80 and LyzM, but not CX3CR1.

### The majority of CSF1R+ splenic red pulp macrophages are labeled with Mac3 and F4/80, but do not express CX3CR1

**Figure 3:**
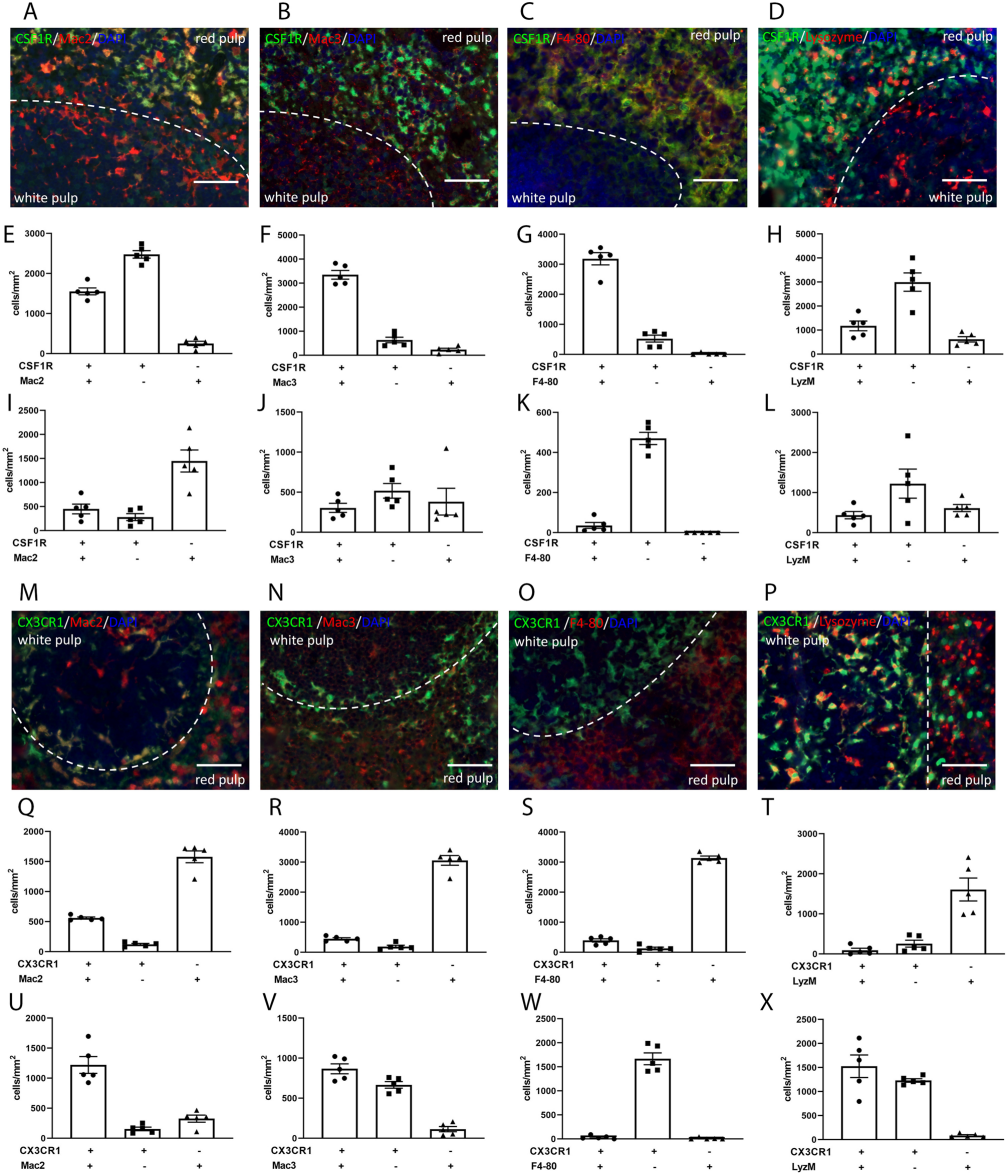
Use of reporter lines and antibodies to identify splenic macrophages in the red and white pulp. (**A**–**D**): Representative images show staining of CSFR1+ macrophages with Mac2, Mac3, F4/80 and anti-lysozyme M antibodies in the red and white pulp. The dotted line represents the marginal zone. (**E**–**H**) Quantitative analysis shows that abundant CSF1R+ cells are located in the red pulp of the spleen. Dual immunofluorescent staining showed that the majority of the CSF1R+ cells in the red pulp are Mac3+ (F, 83.91% ± 3.002) and F4/80+ (G, 85.77% ± 3.117) positive. In contrast, only 38.52% ± 1.449 of CSF1R+ cells are labeled with Mac2 (**E**) and 28.90% ± 5.995 are Lysozyme M+ (stained with anti-lysozyme antibody, clone EPR2994(2)) (**H**). I-L: CSF1R/Mac2 (**I**), CSF1R/Mac3 (J), CSF1R/F4-80 (**K**) and CSF1R/Lysozyme (**L**) staining shows a low density of CSF1R+ cells in the white pulp. (**M**–**P**) Representative images show staining of CX3CR1+ macrophages with Mac2, Mac3, F4/80 and anti-lysozyme M antibodies in the red and white pulp. The dotted line represents the marginal zone. Q-T: Quantitative analysis shows that most of the cells stained with macrophage antibodies in the red pulp are CX3CR1 negative. U-X: Abundant CX3CR1+ cells are located in the white pulp. Mac2 labels most of the CX3CR1+ cells (88.62% ± 2.087, U), whereas Mac3 (V) and Lysozyme M (X) label more than half of the CX3CR1+ cells. F4/80 does not stain any CSF1R+ (K), or CX3CR1+ (W) cells in the white pulp of the spleen. Scale bar: 50 μm.

The splenic red pulp has abundant CSF1R+ macrophages, but also undifferentiated CSF1R-expressing monocytes that can be mobilized upon injury^16,38^ (Fig. 3A–H). The majority of CSF1R+ red pulp macrophages also express Mac3 (Fig. 3B,F) and F4/80 (Fig. 3C,G). In contrast, only 38.5% ± 1.4 of the red pulp CSF1R+ macrophages are positive for Mac2 (Fig. 3A,E) and only 28.9% ± 6.0 express LyzM (Fig. 3D,H). The population of CX3CR1+ cells in the red pulp is much smaller than the abundant CSF1R+ cells (Fig. 3M–T). The vast majority of the CX3CR1+ cells are not labeled with any of the macrophage antibodies (Fig. 3M–T), likely representing “reservoir monocytes”^38,39^.

###  The splenic white pulp contains abundant CX3CR1+ cells, the majority of which express Mac2, Mac3 and LyzM, but not F4/80

The density of CSF1R+ cells in the white pulp is low (Fig. 3A–D,I–L). Although 62.5% ± 2.9 of the CSF1R+ white pulp macrophages express Mac2 (Fig. 3I), the majority are negative for Mac3, F4/80 and LyzM (Fig. 3J–L). Thus, these cells may not be myeloid cells, and may represent B cell progenitors^40^. On the other hand, the white pulp contains a large population of CX3CR1+ cells (Fig. 3M–P,U–X). Virtually all these cells are F4/80-negative (Fig. 3O,W). However, the majority of white pulp CX3CR1+ cells express Mac2, Mac3 and LyzM (Fig. 3U,V,X).

### The majority of CSF1R+ pulmonary macrophages express Mac2 and Mac3

**Figure 4:**
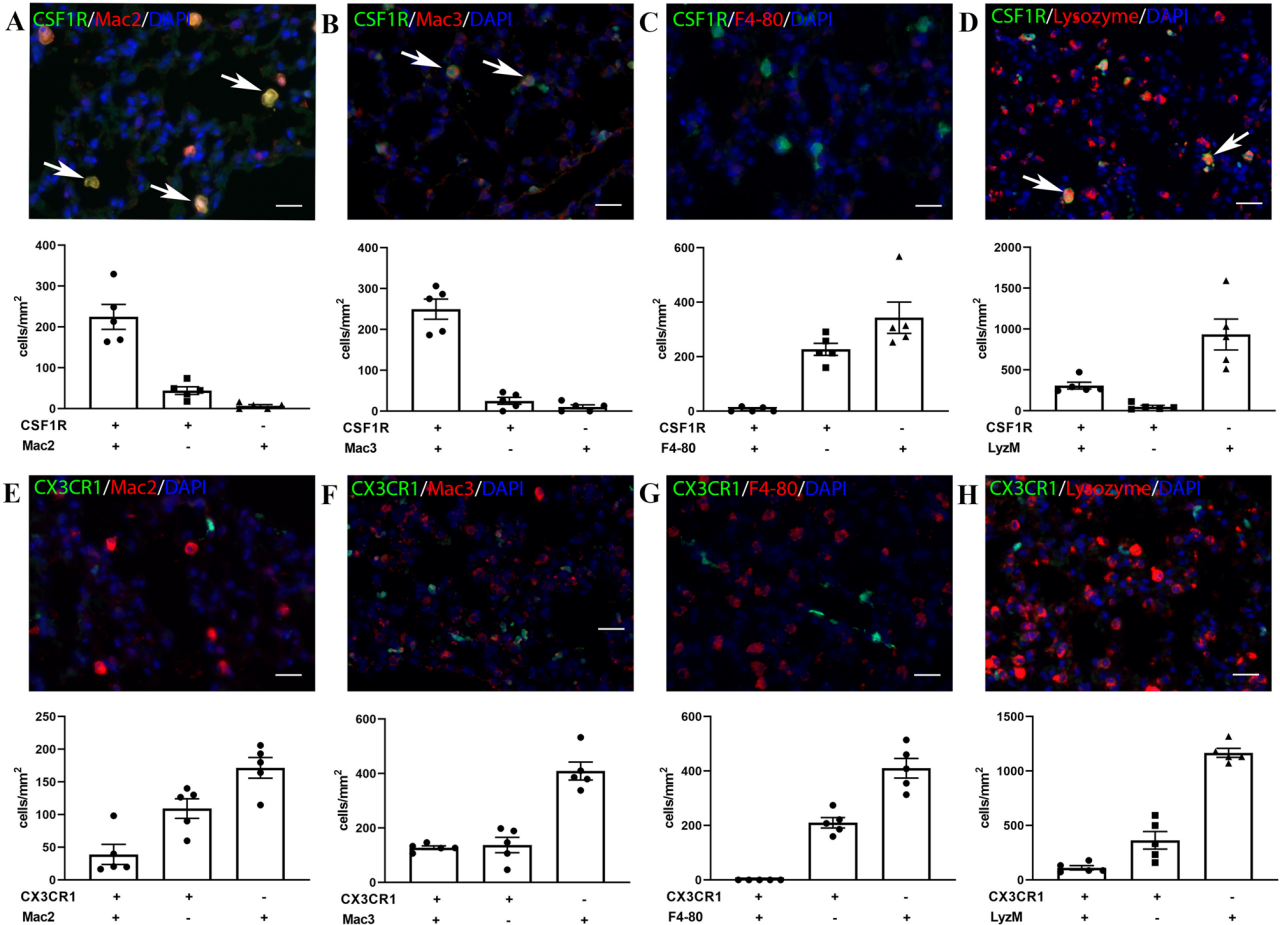
Identification of macrophages in the lung using reporter lines and macrophage antibodies. A-D: Dual fluorescence for GFP and macrophage antibodies in lung sections from CSF1R-EGFP mice. Most CSF1R+ cells are labeled with Mac2 (**A**), Mac3 (**B**), and anti-Lysozyme antibody, clone EPR2994(2) (**D**), but not with the antibody to F4-80 (**C**). E–H: Dual fluorescence for GFP and macrophage antibodies in lung sections from CX3CR1^GFP^ mice. CX3CR1 has limited co-localization with Mac2 (**E**) and Mac3 (**F**), and lysozyme M (**H**). Please note that a significant population of F4/80+ cells is noted in the pulmonary parenchyma; however, these cells do not express CSF1R (**C**) and CX3CR1 (**G**). Scale bar: 20 μm.

A large population of CSF1R+ cells is noted in the lung parenchyma (Fig. 4A–D). The majority of the CSF1R+ cells express Mac2 (Fig. 4A), Mac3 (Fig. 4B), and LyzM (Fig. 4D). In contrast, virtually all CSF1R+ cells in the lung are negative for F4/80 (Fig. 4C). A large population of F4/80+ cells is also noted in the lung; however, this population is independent of the CSF1R+ /Mac2+/Mac3+ cells (Fig. 4C). The CX3CR1 reporter labels a smaller population of pulmonary cells than the CSF1R reporter. The majority of the CX3CR1+ cells are Mac2, Mac3 and LyzM-negative, and all are F4/80-negative (Fig. 4E–H). Thus, the population of CSF1R+ myeloid cells in the lung is distinct from the CX3CR1+ population.

### Mac2, Mac3 and LyzM label a subset of renal interstitial macrophages, but also intensely stain the tubular epithelium

**Figure 5:**
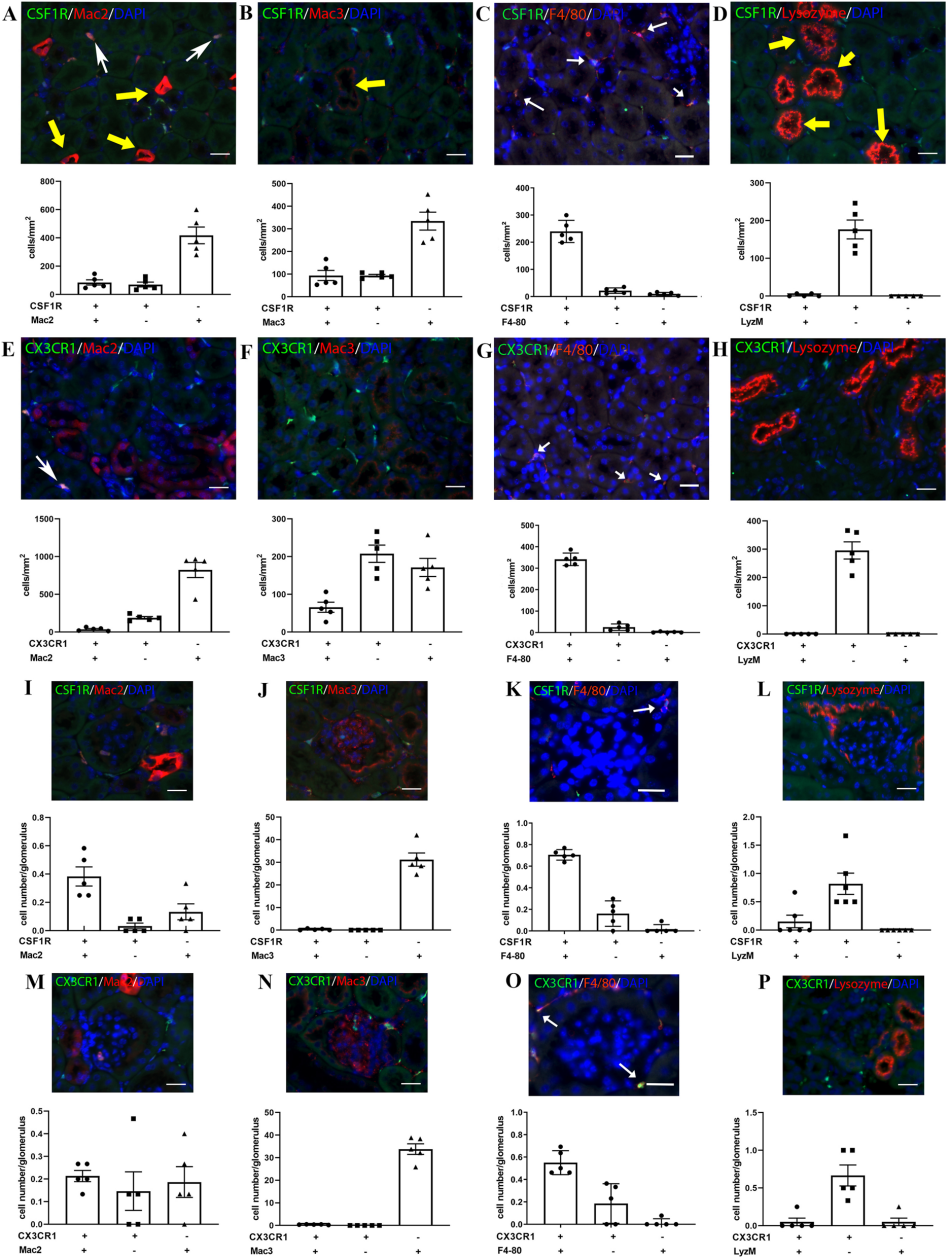
Identification of interstitial and glomerular renal macrophages using reporter lines and antibodies. (**A**–**D**) Dual fluorescence for GFP and macrophage antibodies in kidney sections from CSF1R-EGFP mice. In the tubulointerstititial space ~ 50% of CSF1R+ cells (white arrows) are identified by Mac2 (**A**) and Mac3 antibodies (**B**). Moreover, virtually all CSF1R+ cells are stained for F4/80 (CI:A3-1 clone) (**C**, arrows). F4/80 BM8 clone also identifies renal macrophages; in contrast no staining was obtained with ab 111,101 (Supplemental Fig. I). CSF1R+ renal macrophages were not labeled with the anti-lysozyme antibody clone EPR2994(2) (**D**). A subset of tubular epithelial cells shows intense staining for Mac 2 (**A**, yellow arrows) and lysozyme M (yellow arrows, **D**). The antibody to Mac3 stains the brush-border of the epithelial cells (**B**, yellow arrows). (**E**–**H**) Dual fluorescence for GFP and macrophage antibodies in kidney sections from CX3CR1^GFP^ mice. A small fraction of CX3CR1+ cells express Mac2+ (**E**) or Mac3+ (**F**). Virtually all CX3CR1+ cells stain for F4/80 (clone CI:A3-1) (**G**, arrows). CX3CR1+ cells do not stain with the lysozyme antibody, which labels intensely the tubular epithelium (**H**). I-P: Images illustrating staining of glomerular macrophages. In glomeruli, few CSF1R+ (I-L) and CX3CR1+ cells (**M**–**P**) are noted. The majority of these cells are labeled with Mac2 (**I**, **M**), and F4/80 CI:A3-1 clone (**K**, **O**—arrows), but not with Mac3 (**J**, **N**), and anti-Lysozyme antibodies (**L**, **P**). Scale bar: 20 μm.

In the kidney, most CSF1R+ and CX3CR1+ cells are located in the tubulointerstitium (Fig. 5). 54.8% ± 9.5 of CSF1R+ interstitial cells are labeled with Mac2 (Fig. 5A) and 47.6% ± 6.6 are Mac3+ (Fig. 5B). Staining for F4/80 using the rat monoclonal antibody ab111101 failed to label any of the CSF1R+ cells in the renal interstitium (Supplemental Fig. IA). In order to examine whether the absence of immunoreactivity is due to low sensitivity of the ab111101 antibody, we tested 2 additional antibodies that have been previously used to label renal macrophages in tissue sections (Supplemental Table I). Both the BM8 and CI:A3-1 F4/80 antibodies stained the majority of CSF1R+ renal interstitial macrophages (Supplemental Fig. IB-D). Quantitative analysis, performed using sections stained with the CI:A3-1 clone, showed that the majority of CSF1R+ interstitial cells are F4/80-positive (Fig. 5C). In contrast, staining with the anti-lysozyme antibody did not label renal macrophages (Fig. 5D). Moreover, Mac2, Mac3 and LyzM exhibit cross-reactivity with tubular epithelial cells. Mac2 intensely stains the cytoplasm of a subset of tubular epithelial cells (Fig. 5A), whereas Mac3 and LyzM show weaker labeling of the luminal surface of a significant subpopulation of tubular epithelial cells (Fig. 5B,D).

The density of CX3CR1+ tubulointerstitial cells was similar to that of CSF1R+ cells (Fig. 5E–H). CX3CR1+ cells are F4/80+ (Fig. 5G), whereas only a small fraction of CX3CR1+ cells are Mac2+ or Mac3+ (Fig. 5E,F) and virtually none stain for lysozyme (Fig. 5H). Very few CSF1R+ and CX3CR1+ cells are noted in glomeruli (Fig. 5I–P). The majority of these scarce glomerular macrophages express Mac2 (Fig. 5I,M), and F4/80 (Fig. 5K,O). However, these glomerular macrophages are not labeled with Mac3, or lysozyme M antibodies (Fig. 5J,N and L,P).

### Anti-macrophage antibodies label only a subset of CSF1R+ and CX3CR1+ myocardial cells

**Figure 6:**
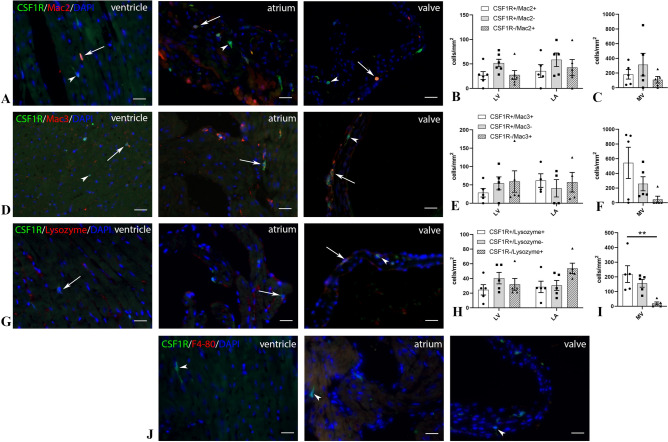
Identification of macrophages in the normal heart using CSF1R-EGFP reporter line and macrophage antibodies. Dual fluorescence for GFP and macrophage antibodies in heart sections from CSF1R-EGFP mice. CSF1R+ cells are sparsely distributed throughout the left ventricle (LV), and left atrium (LA), but exhibit a higher density in mitral valve leaflets. Only a small fraction of CSF1R+ cells express Mac2 (**A**–**C**), Mac3 (**D**–**F**), or LyzM (stained with anti-lysozyme antibody, clone EPR2994(2)) (**G**–**I**) (arrows). F4-80 (ab111101) does not stain any myocardial cells (**J**). Scale bar: 20 μm.

CSF1R+ cells and CX3CR1+ cells are sparsely distributed throughout the left ventricle and the left atrium (Fig. 6, Supplemental Fig. IΙ). The density of CSF1R+ and CX3CR1+ cells in the mitral valve was higher than the corresponding density in the ventricular or atrial myocardium (Fig. 6C, Supplemental Fig. IΙC). Dual immunofluorescence showed that only a fraction of CSF1R+ and CX3CR1+ myocardial cells are labeled with Mac2 (Fig. 6A–C, Supplemental Fig. IΙ A-C), Mac3 (Fig. 6D–F, Supplemental Fig. IΙ D-F) and LyzM (Fig. 6G–I, Supplemental Fig. I G–I). Staining for F4/80 using the recombinant monoclonal antibody ab111101 failed to label any CSF1R+ or CX3CR1+ myocardial cells (Fig. 6J, Supplemental Fig. IIJ). In order to examine whether the absence of immunoreactivity is due to limited sensitivity of the ab111101 antibody, we tested 2 additional anti-F4/80 antibodies (clones BM8 and CI:A3-1) that have been previously used to label cardiac macrophages in tissue sections (Supplemental Table II). None of the F4-80 antibodies stained any myocardial cells (Supplemental Fig. IIIA-B,D-E). Thus, the majority of myocardial CSF1R+ and CX3CR1+ cells are not labeled with anti-macrophage antibodies in paraffin-embedded sections.

### CSF1R+ and CX3CR1+ cells infiltrate the infarcted myocardium

**Figure 7:**
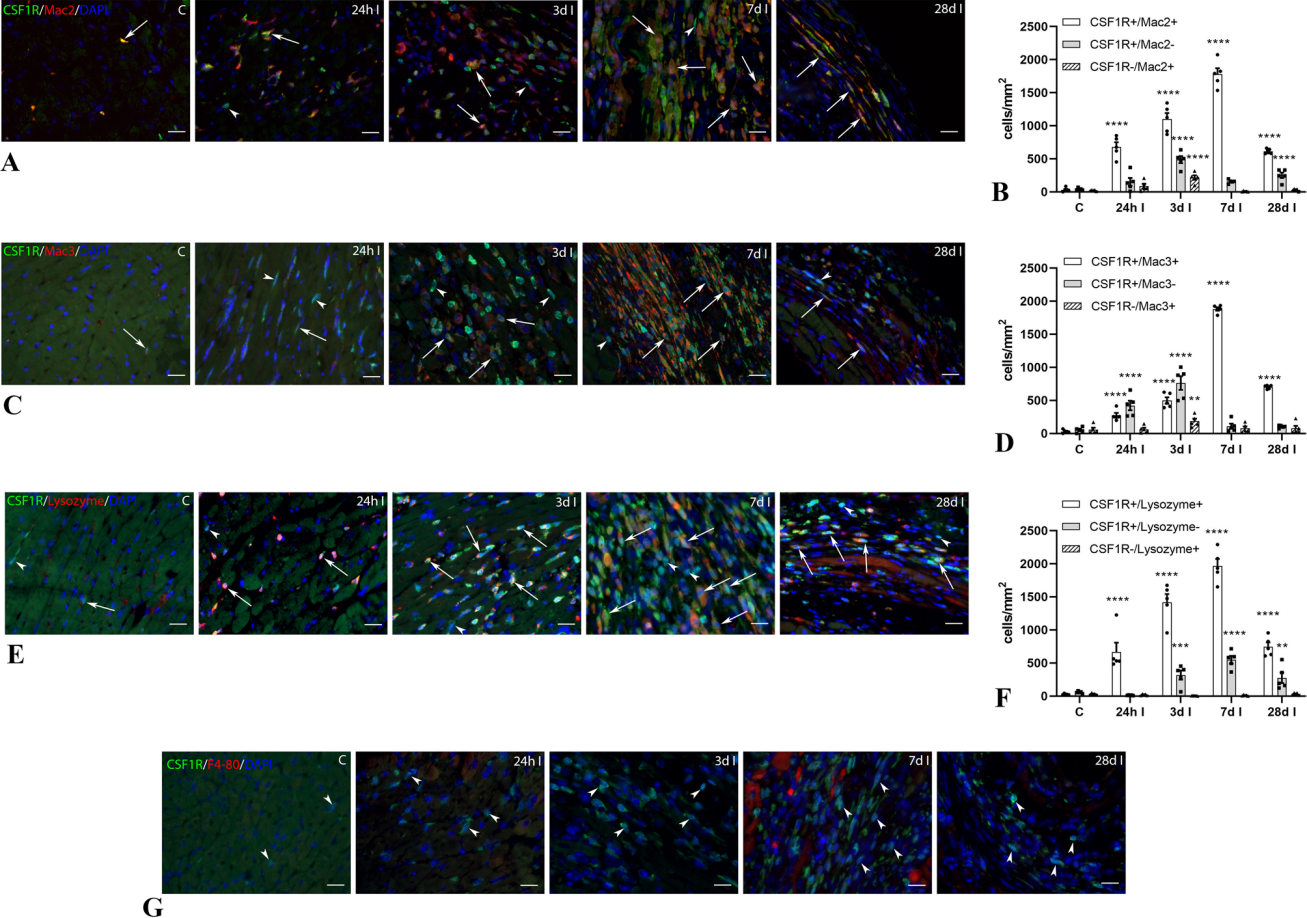
Specificity and staining patterns of macrophage markers during the phases of cardiac repair. CSF1R-EGFP reporter mice underwent non-reperfused myocardial infarction protocols. Dual immunofluorescent staining for GFP and macrophage antibodies (Mac2, Mac3, F4/80 ab111101, Lysozyme antibody clone EPR2994(2)) was performed to identify cardiac macrophages and to evaluate the sensitivity and specificity of various markers. Control (C) mouse hearts have a small population of CSF1R+ cells (arrows). Quantitative analysis shows that the density of CSF1R+ cells in the infarcted myocardium significantly increased after 24 h, and peaked after 7 days of permanent coronary occlusion. No significant increase in the density of CSF1R+ cells was noted in non-infarcted remodeling segments. (**A**,**B**) Although at the peak of the proliferative phase (7d), 99.6% of CSF1R+ cells were labeled with Mac2 (arrows), significant populations of CSF1R+ /Mac2- cells were noted during the inflammatory (24 h–3d) and maturation phase (28d) (arrowheads). (**C**,**D**) Mac3 also stained ~ 90% of CSF1R+ cells at the 7-day timepoint (arrows). However, during the inflammatory phase of cardiac repair (24 h-3d), many CSF1R+ /Mac3- cells were noted (arrowheads), and only ~ 40% of CSF1R+ cells were Mac3 positive. (**E**,**F**) On the other hand, LyzM staining was more sensitive during the inflammatory phase with 98.1% of CSF1R+ cells expressing LyzM at the 24 h timepoint (arrows). Significant populations of LyzM-negative CSF1R+ cells (arrowheads) emerged during the proliferative and maturation phase. G. F4/80 staining was absent in CSF1R+ cells (arrowheads) infiltrating the infarcted myocardium. (**p < 0.01, ***p < 0.001, ****p < 0.0001 vs. corresponding control, n = 4–5/group). Scale bar: 20 μm.

In order to examine the time course of macrophage infiltration in the infarcted myocardium during the various stages of cardiac injury and repair, we used CSF1R-EGFP and CX3CR1^GFP^ reporter mice in a model of non-reperfused myocardial infarction. Four different timepoints were studied, reflecting the early and late inflammatory phase (24 h and 3 days respectively), the proliferative phase (7 days) and the maturation phase of infarct healing (28 days)^41,42^. Immunofluorescence staining demonstrated a rapid increase in macrophage density in the infarcted myocardium, peaking at 7 days post-infarction; in contrast, the number of CSF1R+ and CX3CR1+ cells in the non-infarcted remodeling myocardium did not significantly increase (Fig. 7, Supplemental Fig. IV). CSF1R+ cells were first identified in the infarcted myocardium 24 h after coronary occlusion and peaked at 7 days post-infarction (Fig. 7). In contrast, CX3CR1+ cells exhibited a late infiltration as the number of positive cells increased significantly after 3 days of coronary occlusion (Supplemental Fig. IV).

### Mac2 and Mac3 label most CSF1R+ cells during the proliferative phase of infarct healing, whereas LyzM identifies the CSF1R+ myeloid cells during the inflammatory phase

Anti-macrophage antibodies are routinely used to quantitatively assess macrophage infiltration in infarcted mouse hearts. We used dual immunofluorescence in infarcted CSF1R-EGFP and CX3CR1^GFP^ reporter mice, combining GFP staining and macrophage marker (Mac2, Mac3, F4-80, lysozyme) labeling, in order to evaluate the sensitivity and specificity of these antibodies in macrophage identification. 4 different timepoints (24 h, 3d, 7d, 28d) were studied in order to validate each antibody in identification of macrophages undergoing dynamic phenotypic transitions during the inflammatory, proliferative and maturation phase of infarct healing.

The density of Mac2+ cells was markedly increased in the infarcted myocardium 24 h after coronary occlusion, and peaked after 7 days (Fig. 7A,B). At all timepoints studied, the majority of the Mac2+ cells were also positive for CSF1R (24 h: 89.3% ± 3.4; 3d: 83.6% ± 2.3; 7d: 99.6% ± 0.2; 28d: 95.6% ± 1.2). Thus, although at the peak of the proliferative phase, virtually all CSF1R+ cells were Mac2+, during the inflammatory and maturation phase there was a significant population of Mac2+ cells that did not express CSF1R.

The density of Mac3+ cells was also markedly increased in the infarcted myocardium (Fig. 7C,D), peaking after 7 days of coronary occlusion, and showing a similar time course with Mac2 labeling. The majority of CSF1R+ cells did not stain for Mac3 after 24 h and 3d of reperfusion, likely reflecting lower sensitivity of Mac3 for newly-recruited myeloid cells (neutrophils and monocytes). In contrast, much like Mac2, Mac3 labeled the vast majority of CSF1R+ cells during the proliferative and maturation phase of infarct healing (7 and 28 days after coronary occlusion respectively).

The density of lyzM+ cells was also increased in the infarcted myocardium, and peaked 7 days after coronary occlusion (Fig. 7E,F). In contrast to Mac2 and Mac3, LyzM had an optimal effectiveness in staining CSF1R+ cells at the early timepoint (24 h), likely reflecting co-expression of LyzM and CSF1R (but not Mac2 or Mac3) in neutrophils. A significant CSF1R+ /LyzM- population emerged at the 7- and 28-day timepoints (Fig. 7F), again contrasting the optimal effectiveness of Mac2 and Mac3 in identification of CSF1R+ cells at this stage of healing. This finding may reflect the previously reported emergence of LyzM-negative macrophages in granulomatous inflammation that has been attributed to IL-4 and IL-13 responses^43^. The F4/80 antibody ab111101 did not label any of the CSF1R+ cells in the healing infarct (Fig. 7G). Moreover, the BM8 and CI:A3-1 clones also failed to label the vast majority of infarct macrophages (Supplemental Fig. IIIC,F).

The density of CX3CR1+ cells (Supplemental Fig. IV) was much lower than that of CSF1R+ cells at all timepoints studied, with much less overlap with Mac2 (Supplemental Fig. IV A-B), Mac3 (Supplemental Fig. IV C-D), and LyzM (Supplemental Fig. IV E–F), likely reflecting the lower sensitivity of CX3CR1 as a macrophage marker. The majority of Mac2+ and LyzM+ cells infiltrating the infarcted heart at the 24 h and 3-day timepoints were CX3CR1-negative. Moreover, populations of CX3CR1+ cells that were not labeled by the anti-macrophage antibodies (Supplemental Fig. IV B,D,F) emerged at late timepoints (7–28 days after coronary occlusion).

### Anti-macrophage antibodies label a fraction of fibroblasts in normal tissues

Labeling of many CSF1R-negative and CX3CR1-negative cells by the anti-macrophage antibodies raises concerns regarding the specificity of these markers for macrophages. Because published evidence has suggested that anti-macrophage antibodies may also label fibroblasts, we used the fibroblast-specific PDGFR$\mathrm{\alpha }$-EGFP reporter line to examine whether macrophage markers cross-react with fibroblasts. In normal tissues, a small percentage (< 12%) of the cells labeled with the anti-macrophage antibodies Mac2, Mac3 and F4/80, or with the myeloid cell marker LyzM were identified as PDGFR$\mathrm{\alpha }$+ fibroblasts (Table 1) (Supplemental Fig. V-VIII). In most tissues, LyzM had the lowest level of cross-reactivity with fibroblasts.

**Table 1:** Percentage of PDGFR$\mathrm{\alpha }$+ fibroblasts labeled by macrophage-specific antibodies.

Organ	Mac2	Mac3	Lysozyme (clone EPR2994(2))	F4/80 (ab111101)
Liver	6.66 ± 0.68%	N/A	4.52 ± 1.07%	6.04 ± 0.34%
Spleen (white pulp)	10.94 ± 2.94%	5.34 ± 0.23%	4.90 ± 0.67%	0.00%
Spleen (red pulp)	6.80 ± 1.72%	5.61 ± 0.59%	3.78 ± 0.94%	8.58 ± 0.79%
Lung	3.43 ± 0.70%	2.51 ± 0.48%	3.95 ± 0.47%	4.56 ± 0.81%
Kidney	0.00%	10.55 ± 0.96%	0.00%	N/A
Heart	9.92 ± 1.39%	11.55 ± 1.61%	2.97 ± 1.33%	N/A

### In the infarcted heart, the number of fibroblasts expressing macrophage markers increases during the proliferative phase of repair

**Figure 8:**
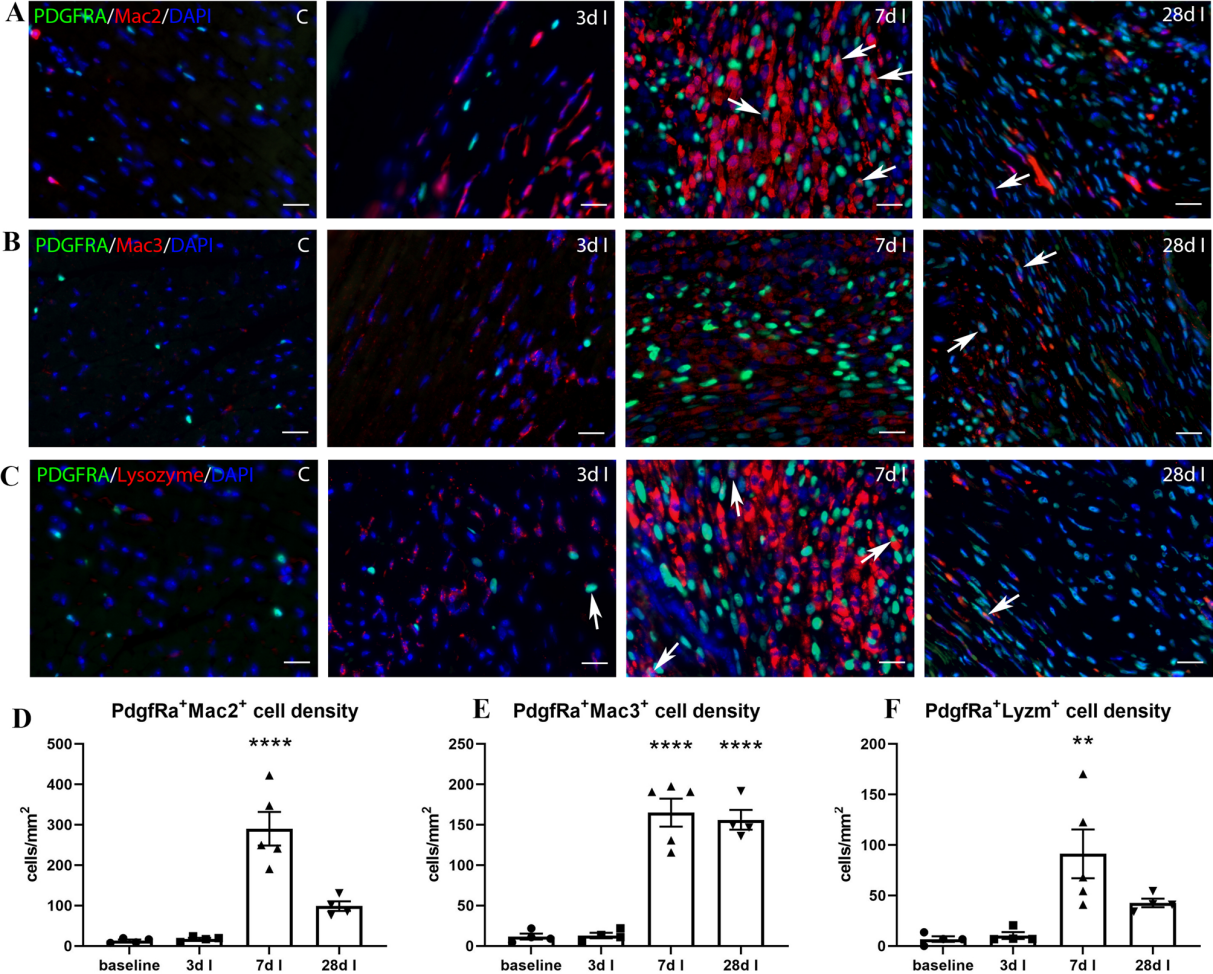
A population of PDGFRα+ fibroblasts, expressing the macrophage markers Mac2, Mac3, and Lysozyme-M emerges during the proliferative phase of infarct healing. PDGFRα-EGFP fibroblast reporter mice underwent non-reperfused myocardial infarction protocols. Dual immunofluorescence for GFP and macrophage antibodies (Mac2, Mac3, Lysozyme antibody clone EPR2994(2)) was performed in control hearts and in infarcted myocardial segments from mice undergoing 3-day, 7-day and 28-day coronary occlusion protocols. Control hearts and early infarcted hearts showed negligible numbers of PDGFRα+ fibroblast cells that express macrophage markers (arrows). At the peak of the proliferative phase (7d after coronary occlusion), a significant population of PDGFRα+ fibroblasts were Mac2 positive (**A**,**D**), Mac3 positive (**B**,**E**), or LyzM positive (**C**,**F**).In comparison to Mac2 and Mac3, LyzM labeled a lower number of PDGFR$\mathrm{\alpha }$+ cells (**p < 0.01, ****p < 0.0001 vs. control, n = 4–5/group). Scale bar: 20 μm.

Some studies have suggested that in the infarcted heart, macrophage subpopulations may express significant amounts of collagen^44^, and even acquire characteristics of matrix-synthetic fibroblasts^44,45^. Moreover, infarct fibroblasts have been suggested to exhibit phagocytic properties^46^, thus acquiring features typically associated with macrophages. Thus, macrophage markers may have reduced specificity in the dynamic environment of the infarct, in which cells exhibit remarkable phenotypic plasticity and may undergo transitions to different phenotypes. In order to examine whether in infarcted hearts, macrophage markers also identify fibroblasts, we performed immunofluorescent staining in infarcted PDGFR$\mathrm{\alpha }$-EGFP fibroblast reporter mice. Fibroblasts in the infarcted heart exhibit dynamic phenotypic changes^47^, transitioning from quiescence to a pro-inflammatory and matrix-degrading state (24 h-3d after infarction)^48,49^, subsequently converting to activated myofibroblasts^50^ (3–14d after infarction), before becoming “matrifibrocytes”, specialized fibroblast-like cells that express a unique profile of proteins that may be involved in scar maintenance^51^. In order to examine whether different macrophage antibodies exhibit cross-reactivity with the distinct fibroblast populations that infiltrate the infarct during the phases of cardiac repair, we studied 3 different timepoints: 3d, 7d and 28d after coronary occlusion (Fig. 8). A population of PDGFR$\mathrm{\alpha }$+ cells expressing macrophage markers emerged after 7 days of coronary occlusion. Mac2+ /PDGFR$\mathrm{\alpha }$+ cells were more abundant than Mac3+ /PDGFR$\mathrm{\alpha }$+ and LyzM+ /PDGFR$\mathrm{\alpha }$+ cells (Fig. 8D,F). Approximately 12.1% ± 2.7 of Mac2+ cells were identified as fibroblasts at the 7-day timepoint. Expression of macrophage markers persisted during the maturation phase, with a significant population of fibroblasts showing labeling for Mac2 and Mac3 (Fig. 8D,E). In comparison to the other macrophage markers, LyzM showed the lowest level of cross-reactivity with infarct fibroblasts at all timepoints (Fig. 8F).

## Discussion

Specific tools for reliable identification of mouse macrophages in histological sections are critical in order to understand the role of the immune response in homeostasis and disease. Experimental studies have routinely used “macrophage-specific” antibodies to label populations of resident or recruited macrophages in many different experimental conditions. The specificity of these tools has not been systematically investigated. It has been suggested that many anti-macrophage antibodies may also detect fibroblasts; however, this notion was based on experiments using non-specific fibroblast markers, such as Fibroblast-specific protein (FSP)-1^33^, a protein known to be highly expressed in activated macrophages^52,53^. In the current study, we used both macrophage (CSF1R-EGFP and CX3CR1^GFP^) and fibroblast reporter mouse lines (PDGFR$\mathrm{\alpha }$-EGFP), in order to evaluate the sensitivity and specificity of anti-macrophage antibodies in normal mouse tissues and in a model of infarctive myocardial injury. Our findings show organ-specific patterns of macrophage reactivity to various antibodies, and suggest that the specificity of various antibodies is reduced following injury, as a subset of injury-site cells co-express fibroblast and macrophage markers.

### Using CSF1R and CX3CR1 reporter mouse lines for macrophage identification

CSF1R/CD115 is broadly expressed by cells of the monocyte/macrophage lineage^8^. CSF1R levels gradually increase as CFU-M precursor cells differentiate to monocytes and macrophages^10–12^. CSF1R-EGFP mice carry an enhanced green fluorescent protein (EGFP) gene downstream of the *Csf1r* promoter and have been extensively used for identification of macrophage and monocyte cell lineages^13,14,54,55^. On the other hand, CX3CR1 has been used as a “pan-macrophage marker” in some studies ^3,28^, whereas in other investigations, expression levels of CX3CR1 were used to distinguish pro- and anti-inflammatory monocyte and macrophage subsets^56^. Pro-inflammatory, “classical” CCR2+ monocytes express moderately high levels of CX3CR1, whereas CCR2- patrolling monocytes exhibit very high CX3CR1 expression^57^. Thus, the level of monocyte EGFP expression in CX3CR1^EGFP^ mice can be helpful for identification of functionally distinct monocyte subsets. However, some investigations have suggested that EGFP expression in CX3CR1^EGFP^ mice does not necessarily reflect CX3CR1 expression levels^58^, due to the extended half-life of EGFP that may result in fluorescence of cells that ceased to express Cx3cr1 as long as 24 h prior to assessment^22^.

In our study, comparison of the distribution of CSF1R+ and CX3CR1+ populations in normal mouse tissues revealed several important observations. First, liver macrophages do not express CX3CR1^32^, but are strongly positive for CSF1R (Fig. 1). Second, in the spleen the majority of CSF1R+ cells are localized in the red pulp and co-express the macrophage-specific antibody F4/80, whereas most CX3CR1+ cells populate the white pulp and are F4/80-negative (Fig. 2). Third, in all organs studied, CSF1R+ cells were more abundant that the CX3CR1+ cells. Fourth, CX3CR1+ cells and CSF1R+ exhibited distinct patterns of staining with anti-macrophage antibodies. These findings suggest that CSF1R-EGFP and CX3CR1^GFP^ reporters label distinct populations in macrophage-rich organs, and that the CSF1R reporter is preferable for broad identification of monocytes and macrophages, whereas the CX3CR1 reporter is useful as part of a systematic approach to characterize specific subsets.

### The specificity of anti-macrophage antibodies in mouse tissues

Anti-macrophage antibodies have been extensively used for identification of macrophage populations in mouse tissues. In the era of single cell transcriptomics and multi-color flow cytometry, simple approaches to label and identify macrophages in paraffin-embedded histopathological sections remain critically important in understanding the cell biology of disease. Experimental studies in mouse models have used many different antibodies to label macrophages, ranging from myeloid cell markers (such as anti-Lysozyme antibodies) to markers considered “specific” for macrophages (such as Mac2, Mac3, F4/80 and CD68). The sensitivity of these markers in staining macrophages in different organs and states of activation has not been systematically validated. The choice of a specific marker in each study is based on the personal experience of the authors, rather than on a well-validated approach. Moreover, several studies have challenged the specificity of anti-macrophage antibodies in different settings, even raising concerns regarding tools considered highly specific for macrophages, such as F4/80^59^ and CD68^33^. Our study carefully characterized the patterns of anti-macrophage antibody labeling using macrophage and fibroblast reporter lines (Table 2).

**Table 2:** Patterns or macrophage labeling in formalin-fixed paraffin-embedded mouse organs using reporter lines and antibodies.

Organ	CSF1R^EGFP^ (reporter)	CX3CR1^EGFP^ (reporter)	Mac2 (IR)	Mac3 (IR)	F4/80 (IR)	Lysozyme (IR)
Liver	Abundant CSF1R+ cells	No detectable staining	Labels all CSF1R+ cells	Diffuse staining in liver parenchyma	Labels all CSF1R+ cells	Staining is dependent on the antibody used. Ab NBP2-61,118 labels all hepatic macrophages, whereas Aby EPR2994(2) stains a subset of CSF1R+ cells
Spleen (RP)	Abundant CSF1R+ cells	Smaller population of CX3CR1+ cells that is not labeled with anti-macrophage antibodies	Labels a subset of CSF1R+ cells	Labels the majority of CSF1R+ cells	Labels the majority of CSF1R+ cells	Labels a subset of CSF1R+ cells
Spleen (WP)	Low density of CSF1R+ cells	Abundant CX3CR1+ cells	Labels the majority of CSF1R+ cells and the majority of CX3CR1+ cells	Labels a subset of CSF1R+ cells and the majority of CX3CR1+ cells	Labels a subset of CSF1R+ , but not CX3CR1+ cells	Labels a subset of CSF1R+ cells and the majority of CX3CR1+ cells
Kidney (TI)	Low density of CSF1R+ cells	Low density of CX3CR1+ cells	Labels ~ 50% of CSF1R+ cells and a small subset of CX3CR1+ cells. Intensely stains tubular epithelial cells	Labels ~ 50% of CSF1R+ cells and a small subset of CX3CR1+ cells. Stains many tubular epithelial cells	BM8 and CI:A3-1 clones, but not the recombinant monoclonal ab111101, label renal interstitial macrophages	Does not stain CSF1R+ and CX3CR1+ cells. Stains the luminal surface of many tubular epithelial cells
Kidney (Gl)	Very few CSF1R+ cells	Very few CX3CR1+ cells	Labels the majority of CSF1R+ and CX3CR1+ cells	No detectable staining	BM8 and CI:A3-1 clones, but not ab111101, label glomerular macrophages	No detectable staining
Lung	Large population of CSF1R+ cells	Significant population of CX3CR1+ cells	Labels the majority of CSF1R+ cells, but a small subset of CX3CR1+ cells	Labels the majority of CSF1R+ cells, but a small subset of CX3CR1+ cells	Labels a large population of CSF1R- and CX3CR1-negative cells	Labels the majority of CSF1R+ cells, but a small subset of CX3CR1+ cells
Heart (N)	Small population of CSF1R+ cells	Small population of CX3CR1+ cells	Labels a subset of CSF1R+ and CX3CR1+ cells	Labels subsets of CSF1R+ and CX3CR1+ cells	No detectable staining of CSF1R+ and CX3CR1+ cells with F4/80 antibodies	Labels a subset of CSF1R+ and CX3CR1+ cells
Heart (MI)	CSF1R+ cell density increases 24 h after infarction (early inflammatory phase), and peaks 7 days after MI (proliferative phase)	Density of CX3CR1+ cells in the infarct is much lower than the number of CSFR1+ cells at all timepoints, and peaked 7 days after MI	Labels virtually all CSF1R+ cells during the proliferative phase. Labels the majority of CSF1R+ cells during the inflammatory and maturation phase	Labels most CSF1R+ cells during the proliferative and maturation phase. Labels the majority of CSF1R+ cells during the inflammatory phase	F4/80 antibodies do not label the vast majority of infarct macrophages	Labels the majority of CSF1R+ cells during the inflammatory phase. A CSF1R+ /LyzM-population emerges during the proliferative phase

Ab, antibody; IR, immunoreactivity; RP, red pulp; WP, white pulp; TI, tubulointerstitium; Gl, glomerular; N, normal; MI, myocardial infarction.

### Mac2 as a macrophage marker

Mac-2, also known as galectin-3 was first described as a 32kD mouse protein expressed on the surface of thioglycollate-elicited macrophages^60,61^, and is a member of the galectin family of galactose-specific lectins^62^. Mac-2 is expressed in normal peripheral blood monocytes, and its expression level increases dramatically upon monocyte to macrophage differentiation^63^. Moreover, Mac2 is a marker of macrophage activation; thioglycollate-elicited macrophages synthesize 10- to 30-fold more Mac-2 than unstimulated peritoneal macrophage subpopulations. Our findings suggest that antibodies to Mac2 have high sensitivity in labeling macrophages in normal mouse tissues. In the liver and in the lung, Mac2 labeled virtually all CSF1R+ cells (Figs. 2A, 4A), the majority of CSF1R+ cells in the kidney were also Mac2 positive. In contrast, Mac2 stained only 38.5% ± 1.4 of the CSF1R+ cells in the red pulp of the spleen and only 30.8% ± 7.9 of CSF1R+ cardiac macrophages. Specificity of Mac2 in identifying macrophages in normal mouse tissues is limited by its reactivity with subsets of epithelial cells in the kidney (Fig. 5A) and in the bowel^64,65^.

In injury sites, the marked expansion of Mac2+ cells reflects for the most part, recruitment and activation of macrophages. Most Mac2 immunoreactive cells in healing myocardial infarcts were also identified as CSF1R+ macrophages. However, during the proliferative phase of infarct healing, a population of Mac2+ fibroblasts emerged (Fig. 8A,D). Our group has documented induction of galectin-3 in a subset of activated myofibroblasts in the pressure-overloaded heart and demonstrated that Mac2 may also label failing cardiomyocytes. The specificity of this labeling was documented using galectin-3 knockout mice^66^.

### Mac3

Mac-3 was first identified as a mouse macrophage differentiation antigen^67^ and has been used to identify macrophages in several different tissues^68^. In our study, Mac3 was superior to Mac2 in labeling CSF1R+ macrophages in the red pulp of the spleen (Fig. 3B), and stained the majority of CSF1R+ cells in the lung (Fig. 4B). In other tissues, its usefulness was limited by concerns regarding its specificity. In the liver, diffuse staining in hepatocytes (Fig. 2D) makes identification of macrophages challenging^69^. In the kidney, Mac-3 did not label the majority of CSF1R+ cells and was also localized in the tubular epithelium (Fig. 5B). In the infarcted myocardium, Mac3 was a reliable marker for identification of CSF1R+ cells during the proliferative phase of cardiac repair (Fig. 7C,D).

### Lysozyme M, a reliable myeloid cell marker

Antibodies to lysozyme have been extensively used to label myeloid cells in many tissues and experimental models. Moreover, LyzM-Cre drivers are effective and reliable tools for myeloid lineage tracing and targeting. Our study shows that virtually all CSF1R+ cells in the liver (Fig. 2D) and in the lung (Fig. 4D) stained for lysozyme. In the kidney, anti-Lysozyme antibodies also label tubular epithelial cells and are not effective tools for identification of the CSF1R+ interstitial or glomerular macrophage populations (Fig. 5D,L). We also noted that the anti-lysozyme antibody clone EPR2994(2) stained only a fraction of liver macrophages, whereas the antibody NBP2-611,118 was more sensitive, labeling virtually all hepatic CSF1R+ cells (Fig. 2). Organ-specific differences in the intensity of staining with anti-lysozyme antibodies may reflect the amount of lysozyme expressed by the macrophages in each organ and the sensitivity of the antibody used. Analysis of the previously published RNA-sequencing data^70^ from the Open Source Mononuclear Phagocytes Project (Immgen ULI, Accession #GSE122108) showed that expression levels of *Lyz2* the gene encoding lysozyme-M are lower in hepatic, splenic and kidney macrophages (Supplemental Fig. IX), which seem to exhibit reduced immunoreactivity for lysozyme. In the infarcted heart, lysozyme M staining labeled the abundant myeloid cells that infiltrate the infarct during the inflammatory phase of cardiac repair (Fig. 7E,F). However, during the proliferative and maturation phases of infarct healing (7–28 days after coronary occlusion), a significant population of CSF1R+ /LyzM- cells emerged (Fig. 7F); these cells expressed both Mac2 and Mac3. The emergence of LyzM-negative macrophages in healing infarcts may reflect cytokine-mediated downmodulation of lysozyme M^43^.

### Tissue specific staining patterns of F4/80

F4/80 is a 160 kd plasma membrane glycoprotein^71–73^ that has been widely used as a macrophage marker in mice^74^. F4/80 expression has been reported in mature macrophage populations^75^, but is low or even absent in circulating monocytes^76^. Although traditionally considered specific to macrophages, F4/80 has also been found to be expressed on other cell types. F4/80 expression by eosinophils is well-documented^59,77^. Moreover, in models of tissue injury, F4/80 expression has been demonstrated in cells with characteristics of activated myofibroblasts^78^. In our study, we used 3 different F4/80 antibodies: the recombinant rabbit monoclonal anti-F4/80 antibody clone SP115 (Abcam ab111101), and the rat monoclonal antibodies CI:A3-1 (Abcam ab6640) and BM8 (eBioscience). We found organ-specific patterns of F4/80 staining, which are dependent on the specific antibody used. All 3 antibodies identified macrophages in the liver and in the red pulp of the spleen, showing that F4/80 is highly sensitive and specific in identification of CSF1R+ macrophages in these organs (Figs. 2B and 3C). In the kidney, the BM8 and CI:A3-1 clones, but not the clone SP115, identified tubulointerstitial and glomerular macrophages. In contrast, in the lung, F4/80 stained a population of parenchymal cells that were not labeled for CSF1R, CX3CR1, Mac2 or Mac3 (Fig. 4). Moreover, the macrophages in normal and infarcted hearts did not stain with any of the 3 anti-F4/80 antibodies. The organ-specific patterns of F4/80 immunofluorescence in paraffin-embedded sections are surprising and contrast the broad use of F4/80 antibodies to detect and sort macrophages in the lung^79^ and in the myocardium^46,80,81^, using flow cytometry studies. Analysis of the RNA-sequencing data^70^ from the Open Source Mononuclear Phagocytes Project showed that cardiac and pulmonary macrophages exhibit lower levels of *Adgre1* expression (the gene encoding F4/80) than macrophages harvested from the spleen or liver (Supplemental Fig. IX). Thus, the absence of staining of cardiac macrophages with F4/80 antibodies may reflect, at least in part, organ-specific differences in protein expression. Moreover, tissue-specific post-translational modifications of the F4/80 protein^82^ may alter antibody binding, greatly affecting the effectiveness of labeling. Antigen masking during fixation or paraffin embedding may also exhibit organ-specific characteristics, resulting in selective loss of F4/80 immunoreactivity in some tissues.

### Do macrophage markers overlap with fibroblasts in injured tissues?

Studies in many different organs suggest that tissue injury expands the phenotypic diversity of interstitial cells, leading to emergence of new cellular phenotypes and states of activation. Some investigations suggested that following injury, macrophages may acquire characteristics of fibroblasts^83^; however, several other lineage tracing studies have demonstrated that such events may be exceedingly rare, and that injury-site fibroblasts are predominantly derived from resident fibroblast populations^84^. Our study demonstrated that the kidney and the lung harbor significant populations of cells that, despite exhibiting intense staining with macrophage antibodies, lack CSF1R expression. Moreover, during the proliferative phase of infarct healing, a large population of CSF1R-negative Mac2+ and Mac3+ cells infiltrated the infarct zone. We used the well-documented fibroblast-specific PDGFR$\mathrm{\alpha }$ reporter line to examine potential overlap between antibody-labeled macrophages and tissue fibroblasts. Our findings show a marked expansion of PDGFR$\mathrm{\alpha }$+ cells expressing macrophage markers (Mac2, Mac3, and LyzM) at the peak of the proliferative phase of cardiac repair (Fig. 8). These cells may represent macrophages acquiring fibroblast characteristics, fibroblasts exhibiting expression of macrophage proteins, or intermediate states of cell differentiation. In any case, the emergence of these cells adds to the challenges of cell identification in sites of injury.

### Conclusions

Optimal selection of appropriate tools for macrophage identification in mouse studies requires understanding of the organ-specific patterns of expression of various macrophage markers. No single marker is suited for all organs and all states of activation. A combination of approaches using reporter lines and suitable antibodies may be needed for reliable identification of macrophage populations. Moreover, in injured tissues, expansion of the palette of macrophage phenotypes through emergence of cells expressing both macrophage and fibroblast markers further complicates cell identification.

## Materials and methods

### Animals

The study was performed in accordance with the ARRIVE guidelines. Animal studies were approved by the Institutional Animal Care and Use Committee at Albert Einstein College of Medicine and conform with the Guide for the Care and Use of Laboratory Animals published by the National Institutes of Health. We used male and female, 3–4 month of age, “MacGreen” CSF1R-EGFP reporter mice^13^, CX3CR1^GFP^ reporter mice^22^ and PDGFR$\mathrm{\alpha }$-EGFP reporter mice^85,86^ from our own colonies (originally obtained from Jackson labs, stock No: 018549, 005,582, 007,669). Genotyping was performed using standard protocols.

### Tissue harvesting and processing

CSF1R-EGFP, CX3CR1^GFP^and PDGFR$\mathrm{\alpha }$-EGFP mice were sacrificed at 4 months of age (n = 4–5). The heart, spleen, lung, liver and kidney were harvested, fixed in zinc-formalin (Anatech Ltd., Fisher Scientific) and embedded in paraffin for histological studies. Sequential 5 µm sections were cut by microtome.

### Mouse model of non-reperfused myocardial infarction

A model of non-reperfused myocardial infarction induced through coronary ligation was used, as previously described by our group^87^. Female and male mice (CSF1R-EGFP, CX3CR1^GFP^ and PDGFR$\mathrm{\alpha }$-EGFP, n = 4–5/group), 3–4 months of age, were anesthetized using inhaled isoflurane (4% for induction, 2% for maintenance). For analgesia, buprenorphine (0.05–0.2 mg/kg s.c) was administered at the time of surgery and q12h thereafter for 2 days. Additional doses of analgesics were given if the animals appeared to be experiencing pain (based on criteria such as immobility and failure to eat). Intraoperatively, heart rate, respiratory rate and electrocardiogram were continuously monitored and the depth of anesthesia was assessed using the toe pinch method. The left anterior descending coronary artery was occluded for 24 h, 3 days, 7 days, or 28 days. At the end of the experiment, the hearts were harvested, fixed in zinc-formalin and embedded in paraffin for histological studies. Sequential 5 µm sections were cut from base to apex.

### Immunofluorescence staining

For immunofluorescence staining, the paraffin-embedded sections were dewaxed and subjected to antigen retrieval in a steamer for 20 min in citric buffer (pH 6.0) or Tris–EDTA (pH 9.0) buffer followed by cooling at room temperature for 1 h. They were blocked with serum for 1 h at room temperature. Sections were incubated overnight at 4 °C with the following primary antibodies: rabbit anti-lysozyme antibody (1:250; Abcam, ab108508), rabbit anti-lysozyme antibody (1:100; Novus Biologicals, NBP2-61,118), rat anti-Mac2 antibody (1:1000; Cedarlane, CL8942AP), rat anti-CD107b/Mac3 antibody (1:100; BD, 553,322), recombinant rabbit monoclonal anti-F4/80 antibody SP115 (1:100; Abcam, ab111101), rat monoclonal anti-F4/80 antibody clone CI:A3-1 (1:100; Abcam ab6640), rat monoclonal anti-F4/80 clone BM8 (1:100; eBioscience, 14–4801-82), FITC-conjugated goat anti-GFP antibody (1:400; Abcam, ab6662). After washing the slides in PBS, slides were incubated with Alexa Fluor 594 goat anti-rabbit (1:400; A-11012, Thermo Fisher Scientific) or Alexa Fluor 594 goat anti-rat (1:400; A-11007, Thermo Fisher Scientific) secondary antibody for 1 h at room temperature, followed by washes. TrueBlack® Lipofuscin Autofluorescence Quencher (23,007, Biotium) was used to quench autofluorescence before mounting with Fluoro-Gel II Mounting Medium (Electron Microscopy Sciences, 17,985–50). Images were acquired with Zeiss Axio Imager M2 microscope (Zeiss).

### Quantitative analysis of histological endpoints

Quantitative assessment of the density of lysozyme positive cells, Mac2 positive cells, Mac3 positive cells, F4/80 positive cells, GFP positive cells, lysozyme and GFP positive cells, Mac2 and GFP positive cells, Mac3 and GFP positive cells and F4/80 and GFP positive cells was performed by counting the number of cells in at least 5 random fields from 2–3 different sections from each heart, liver, lung, spleen and kidney with the use of ZEN 3.2 software (Zeiss). Cell density was expressed as cells/mm^2^.

### Statistical analysis

Results are presented as mean ± SEM. For comparisons of multiple groups, one-way ANOVA was performed followed by Tukey’s multiple comparison test. The Kruskal–Wallis test, followed by Dunn’s multiple comparison post-test was used when one or more groups did not show Gaussian distribution. Statistical significance was set at 0.05.

## Supplementary Information

Supplementary Information.

## Data Availability

The datasets used and/or analyzed during the current study are available from the corresponding author on reasonable request.
