# Supplementary material for: Identification of macrophages in normal and injured mouse tissues using reporter lines and antibodies
Source: Sci Rep. 2022 Mar 16;12:4542. doi: 10.1038/s41598-022-08278-x (PMC8927419; doi:10.1038/s41598-022-08278-x)
Supplement: Supplementary file 1 — Supplementary Information. [file 41598_2022_8278_MOESM1_ESM.pdf]

SUPPLEMENTAL DATA:

Identification of macrophages in normal and injured mouse tissues using reporter lines and antibodies

Bijun Chen, Ruoshui Li, Akihiko Kubota, Linda Alex, and Nikolaos G Frangogiannis

SUPPLEMENTAL TABLES:

Supplemental Table I: Studies using F4/80 antibodies for immunohistochemical identification of renal macrophages in mice

Strain of mice and pathology studied	Method of tissue processing	Source of antibody	Findings	Ref.
BALB/c mice, control and in an adriamycin nephropathy model	frozen sections	rat anti-mouse F4/80 (BM8; 1:200) antibodies	In normal and diseased kidneys, F4/80+CD11c+ cells were noted in the cortex, whereas F4/80+CD11c− cells were scattered throughout the kidney.	[1]
Adult and fetal CBA/T6T6 male mice, could be generalized to inbred and outbred mice	Perfusion-fixed tissue (0.5% glutaraldehyde), embedded in paraffin.	F4/80 hybridoma secreting a rat IgG2b	F4/80+ macrophages were suggested to constitute the majority of the renal medullary interstitial cell population.	[2]
C57BL/6 mice	Frozen sections	conjugated anti-F4/80 e660 (eBiosciences)<br><br>conjugated anti-F4/80 (clone Cl:A3-1, AbD Serotec)	F4/80bright cells constitute ~ 50% of CD45+ cells in the kidney of C57BL/6 mice.	[3]
mixed background	fixed frozen sections (4% paraformaldehyde)	rat anti-F4/80 (Abcam)	F4/80+ cells reside in the tubulointerstitium	[4]
Genetically manipulated C57BL/6J mice	Fixed frozen sections (paraformaldehyde)	anti-F4/80- (clone BM8),	F4/80+ cells identified in cortex and in medulla.	[5]
C57BL/6J and mixed background mice in a model of acute kidney injury.	Fixed frozen sections (4% paraformaldehyde)	F4/80 (BM8)	F4/80+ macrophages identified in medulla and cortex.	[6]
Genetically targeted mice	Frozen sections	Anti-F4/80 (Rat monoclonal) Bio-Rad (MCA497) Clone CI:A3-1	F4/80+ cells were noted in the cortex and medulla	[7]

Genetically targeted and transgenic mice in BALB/c background. Morel of unilateral ureteral obstruction (UUO).	Paraffin-embedded sections (fixative not indicated) Unmasking performed using 0.05% trypsin and proteinase K (15min at room temperature)	Clone CI:A3-1 (Serotec)	Staining with F4/80 distinguished macrophages from FSP1+ cells (considered to be fibroblasts) in fibrotic tissues	8
Young adult C57BL/6 mice.	Paraffin-embedded (Carnoy's)	Anti-F4/80 antibody (Serotec)	F4/80+ cells were identified in the kidney	9

Ref, Reference

Supplemental Table II: Studies using the F4/80 antibody for immunohistochemical identification of cardiac macrophages in mice

Strain of mice and pathology studied	Method of tissue processing	Source of antibody	Findings	Ref.
Cx3cr1$^{GFP/+}$ mice (10-12 weeks of age, female)	Frozen sections (fixed in 4% paraformaldehyde).	Unclear (however, F4/80 clone BM8 was used for flow cytometry in the same study).	F4/80 co-stained CX3CR1+ cells in normal heart tissue	[10]
Neonatal hearts in the presence or absence of infarction	Paraffin-embedded sections (4% paraformaldehyde).	Anti-F4/80 (Serotec)	F4/80+ macrophages were identified in infarcted neonatal hearts.	[11]
Control and genetically manipulated C57BL/6J mice, in the presence or absence of infarction.	Unknown	rat anti-mouse F4/80 (clone BM8), (from Invitrogen and eBioscience.	F4/80+ macrophages were identified in the infarct and border zone	[12]
CD68-GFP mouse embryos in a C57BL/6J background	cryosections	Anti-F4/80 (clone CI: A3-1)	F4/80 staining was completely absent in the developing heart when compared with CD68-GFP expression.	[13]
Male C57BL/6 mice, in the presence or absence of infarction	Paraffin-embedded sections (formalin-fixed).	anti-F4/80 (eBiosciences)	F4/80+ cells identified in infarcted hearts	[14]
Embryonic, neonatal and adult genetically manipulated mice.	Unclear (both frozen sections and paraffin-embedded sections were used in the study; however, the type of sections used for F4/80 staining is not indicated	Rat monoclonal anti F4/80 antibody (Bio rad Cat# MCA497)	F4/80 staining was noted in hearts after E13.5	[15]
Young male C57BL/6J mice, undergoing sham or transverse aortic constriction (to induce left ventricular pressure overload).	Paraffin-embedded sections. Antigen retrieval was performed with boiling citrate buffered saline	Rat monoclonal anti-F4/80 (Ab6640, Abcam) antibody	F4/80+ cells were identified in both sham and pressure-overloaded hearts	[16]

C57BL/6 mice undergoing sham or myocardial infarction protocols.	Paraffin-embedded (4% paraformaldehyde). Heat-mediated antigen retrieval was performed.	Rat monoclonal anti-F4/80 (ab6640, Abcam)	F4/80+ macrophages noted in the infarcted heart	17
Control and genetically-manipulated C57Bl/6 mice in the presence or absence of myocardial infarction	Paraffin-embedded sections (zinc-formalin was used as a fixative)	Rat anti-mouse F4/80 (Research Diagnostics Inc)	F4/80+ macrophages were noted in the infarcted heart	18
Control and infarcted C57Bl6J mice.	Paraffin-embedded sections (zinc-formalin was used as a fixative)	Rat anti-mouse F4/80 (Research Diagnostics Inc)	F4/80+ macrophages were noted in the infarcted heart during the inflammatory phase. However, F4/80 immunoreactivity was much lower at later timepoints	19

Ref., Reference.

SUPPLEMENTAL FIGURES:

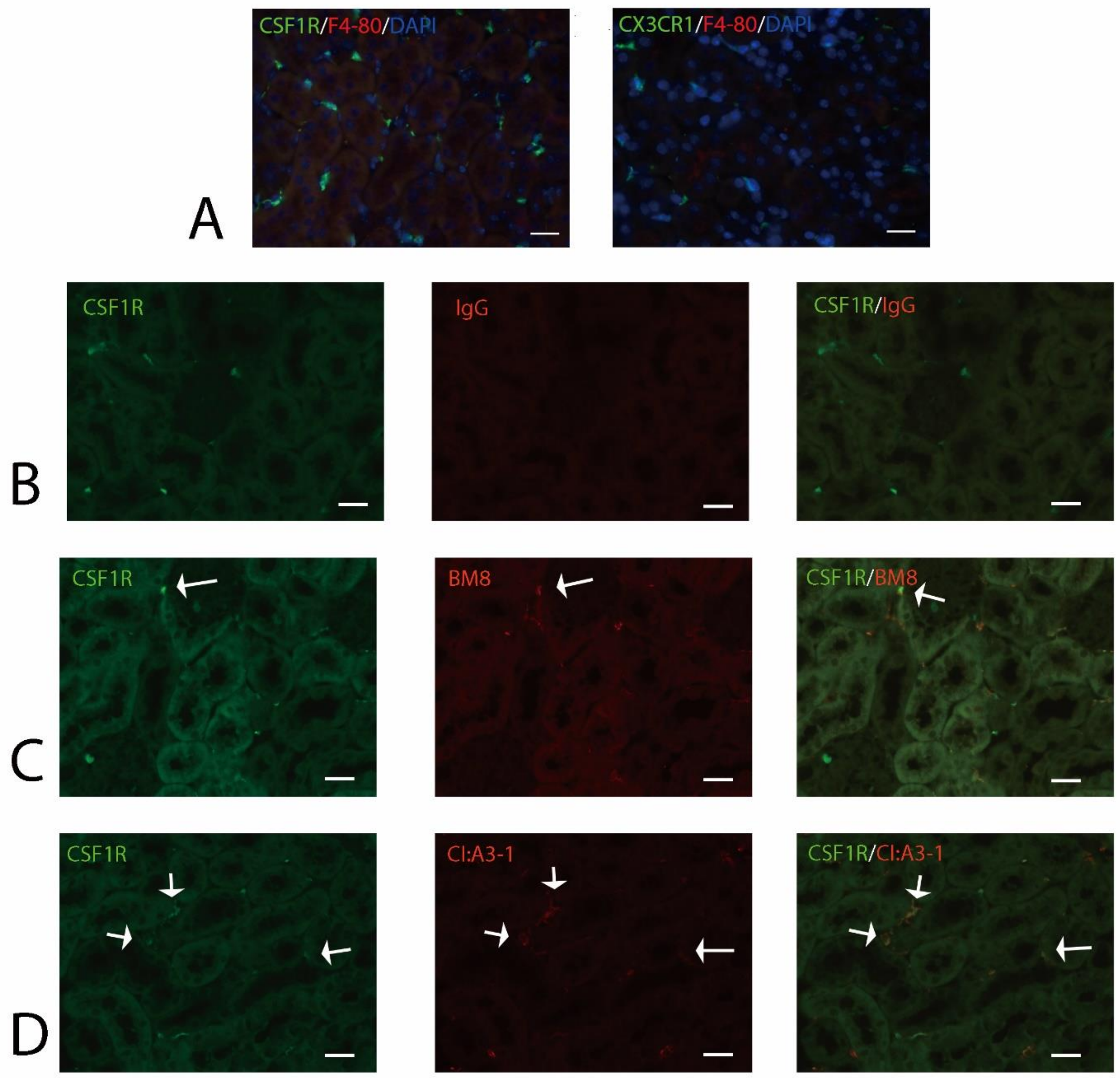

Supplemental Figure I: Use of 3 different anti-F4/80 antibodies to label macrophages in the kidney using sections from CSF1R^{EGFP} mice. A. Staining with the recombinant rabbit monoclonal anti-F4/80 antibodyab111101 showed no staining of CSF1R+ cells in the mouse kidney. However, staining for the BM8 (C) and CI:A3-1 (D) clones, labeled virtually all CSF1R+ renal macrophages (arrows). Panel B shows rabbit IgG-stained controls. Scalebar=20μm.

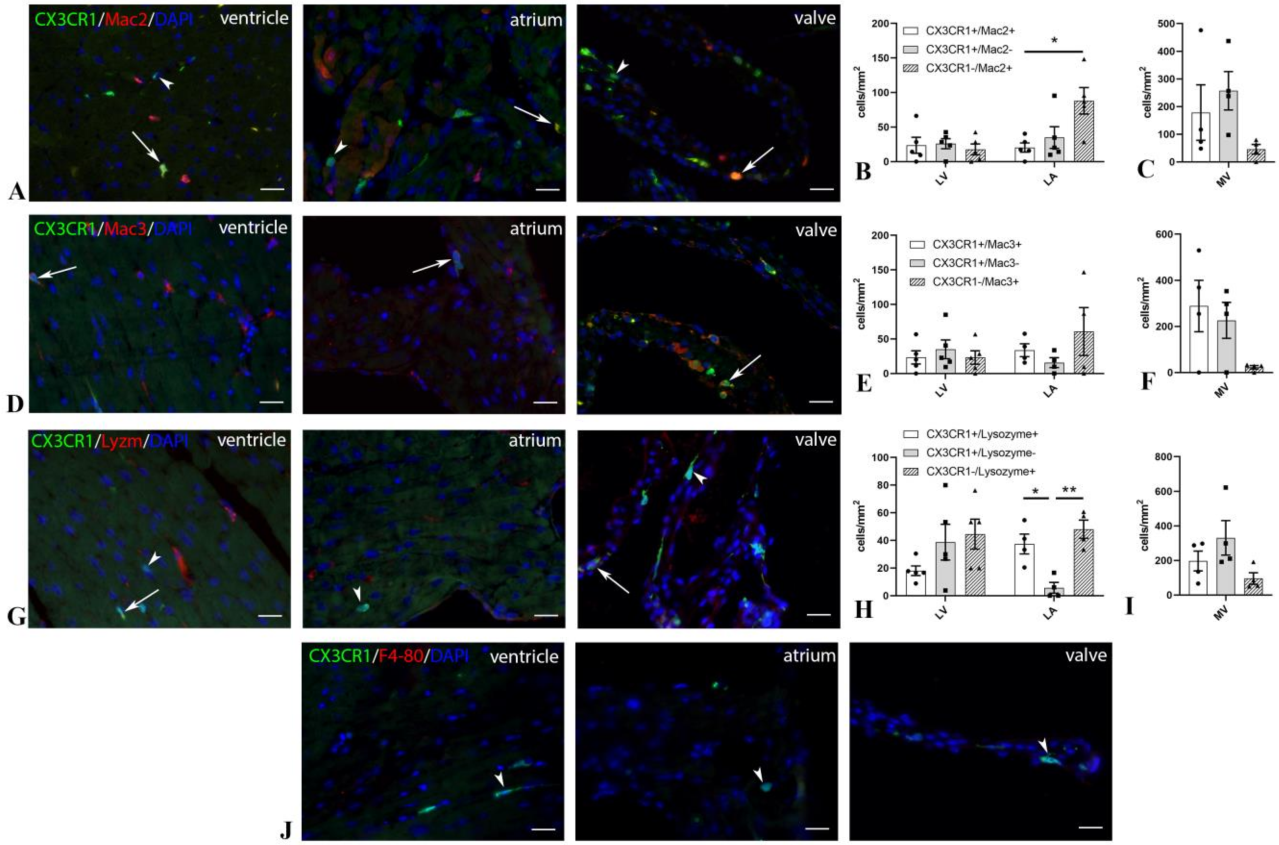

Supplemental Figure II: Identification of macrophages in the normal heart using the CX3CR1^{GFP} reporter line and macrophage antibodies. Dual fluorescence for GFP and macrophage antibodies in heart sections from CX3CR1^{GFP} mice. CX3CR1+ cells are sparsely distributed throughout the left ventricle (LV), left atrium (LA), but exhibited a higher density in mitral valve leaflets. Only a small fraction of CX3CR1+ cells express Mac2+ (A-C), Mac3 (D-F), or LyzM (G-I) (arrows). Anti-F4-80 (ab111101) does not label any myocardial cells (J). Arrowheads point to CX3CR1+ cells that are not labeled with macrophage antibodies (*$p<0.05$, **$p<0.01$, n=5/group). Scale bar: 20 μm.

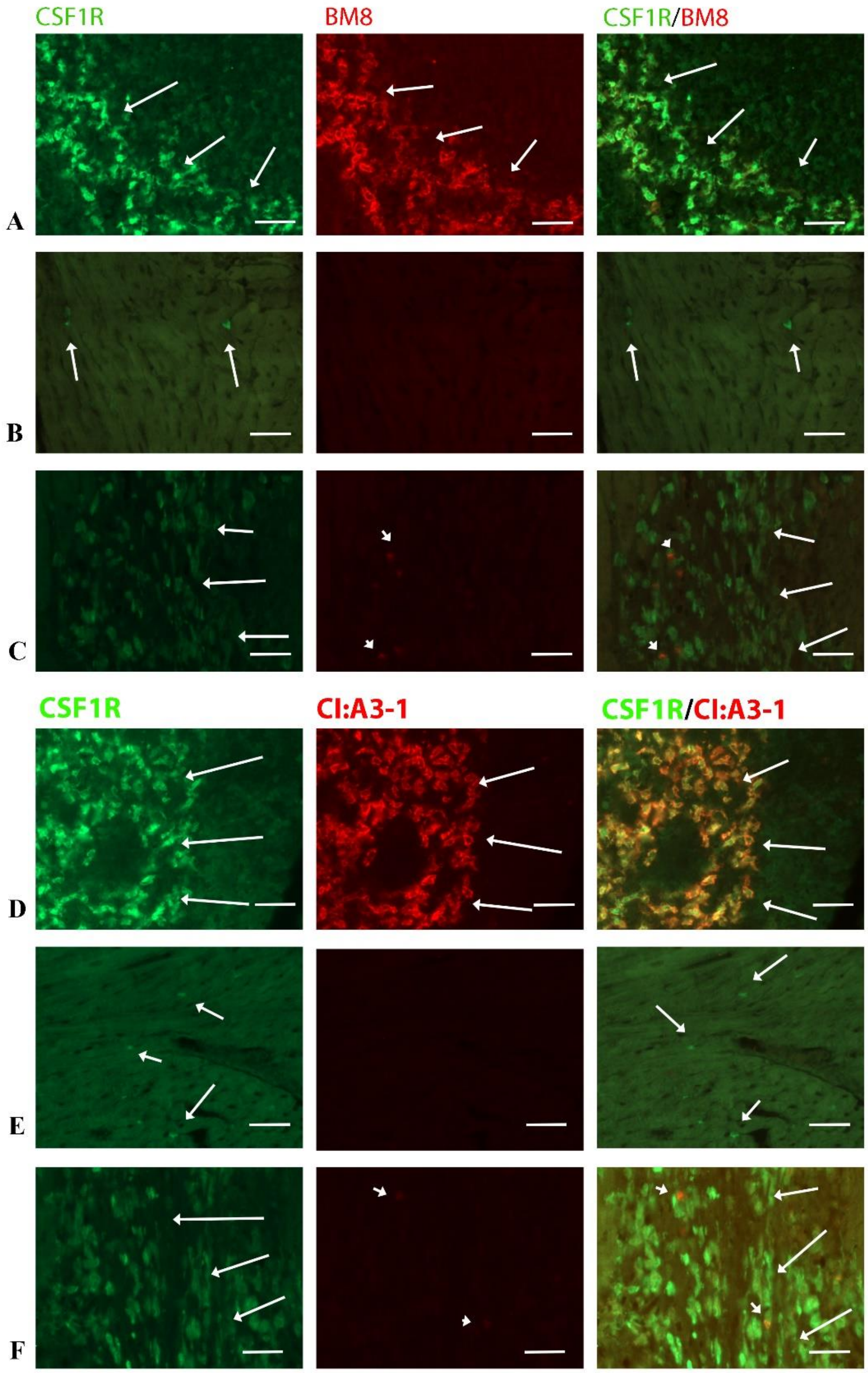

Supplemental Figure III: F4/80 antibodies do not identify macrophages in normal and infarcted hearts. In order to examine whether the absence of staining of cardiac macrophages with the recombinant monoclonal anti-F4/80 antibody ab111101 reflects low sensitivity of the antibody, we used 2 additional antibodies that have been previously used to label macrophages in mouse hearts (Supplemental Table II). The BM8 clone intensely stained CSF1R+ macrophages in the splenic red pulp (A, arrows). However, in the normal myocardium, no staining of CSF1R+

macrophages (arrows) for F4/80 BM8 was noted (B). In the infarcted heart, there was abundant infiltration with CSF1R+ macrophages (long arrows), peaking at the 7-day timepoint. Very few of these cells (short arrows) exhibited anti-F4/80 BM8 staining (C). Similar results were obtained with the CI:A3-1 F4/80 clone. This antibody also intensely stained the macrophages of the splenic red pulp (D, arrows), but did not label cardiac resident CSF1R+ macrophages (E, arrows). In the infarcted heart (F), the vast majority of the abundant CSF1R+ macrophages (long arrows) did not stain for F4/80 CI:A3-1. Very few CSF1R+ cells (short arrows) exhibited anti-F4/80 CI:A3-1 staining. Thus, anti-F4/80 antibodies cannot be used for reliable identification and quantitation of infarct macrophages in paraffin-embedded sections. Scalebar=50μm

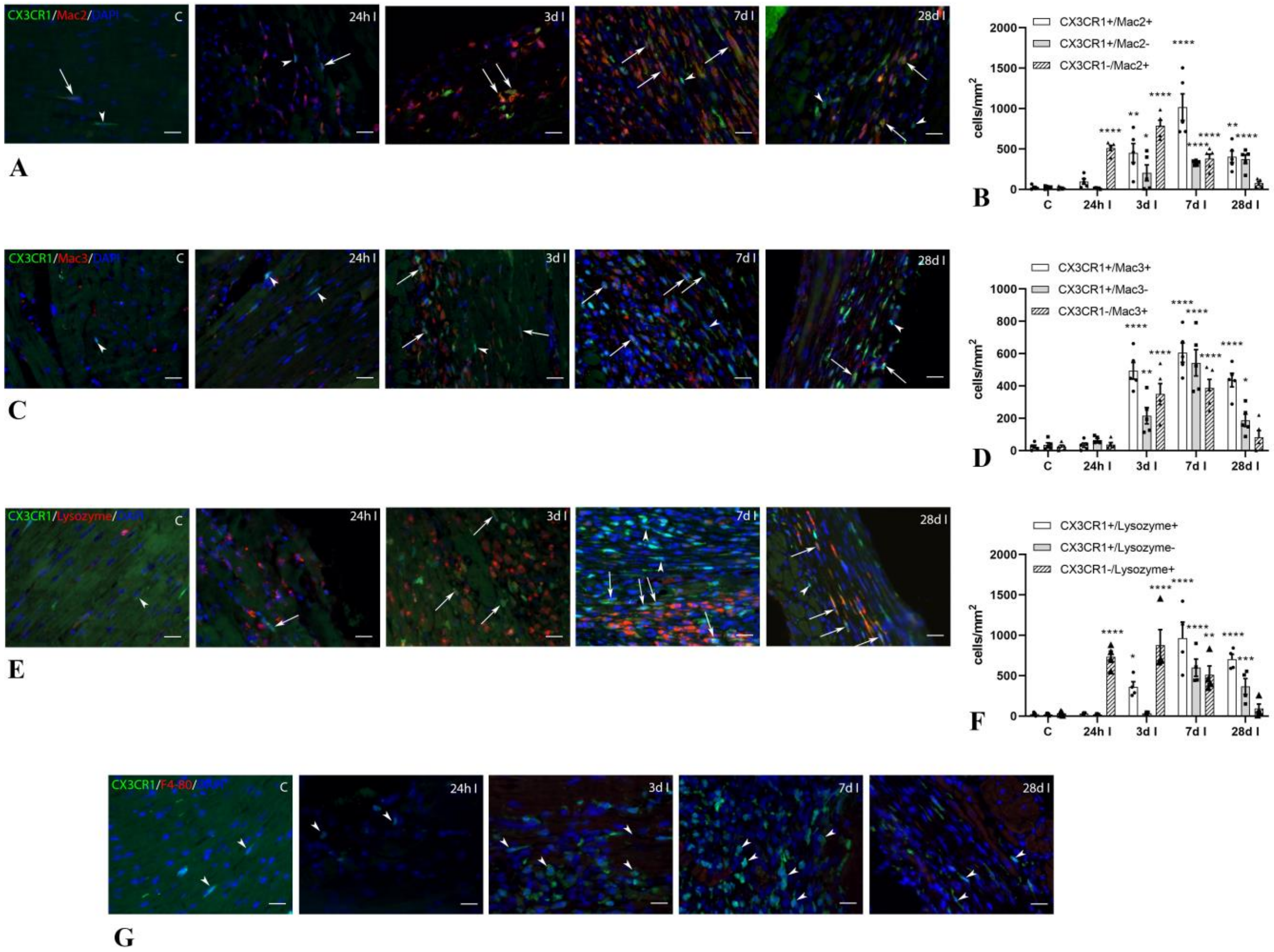

Supplemental figure IV: Time course of CX3CR1+ myeloid cells infiltrating the infarcted myocardium. CX3CR1-GFP reporter mice underwent non-reperfused myocardial infarction protocols. Dual immunofluorescent staining for GFP and macrophage markers (Mac2, Mac3, F4/80, Lysozyme) was used to identify cardiac macrophages. Quantitative analysis showed that the density of CX3CR1+ myeloid cells in the infarcted myocardium significantly increased after 3 days and peaked after 7 days of permanent coronary occlusion. No significant increase in the density of myeloid cells was noted in non-infarcted remodeling segments. The density of CX3CR1+ cells in the infarct zone was lower than that of CSF1R+ cells. The majority of Mac2+ (A-B) and LyzM+ (E-F) cells infiltrating the infarct during the inflammatory phase were CX3CR1-negative. More than half of the CX3CR1+ cells were labeled with Mac2 (A-B) and LyzM (E-F) at all timepoints studied. C-D: Mac3 also stained more than 50% of the CX3CR1+ cells at the 3, 7 and 28-day timepoints Significant number of Mac2 (B), and Lysozyme+ cells (F) were CX3CR-negative in the infarcted myocardium 24h-7days after coronary occlusion. D. Numerous CX3CR1- Mac3+ cells were identified in the infarcted area after 3-7 days of coronary occlusion. G. F4/80 did not stain the CX3CR1+ cells. Arrows show the double positive cells (labeled for both CX3CR1 and the macrophage antibodies), whereas arrowheads point to CX3CR1+ cells that do not stain for the antibody (*p<0.05, **p<0.01, ****p<0.0001 vs. control, n=4-5/group). Scale bar: 20 µm.

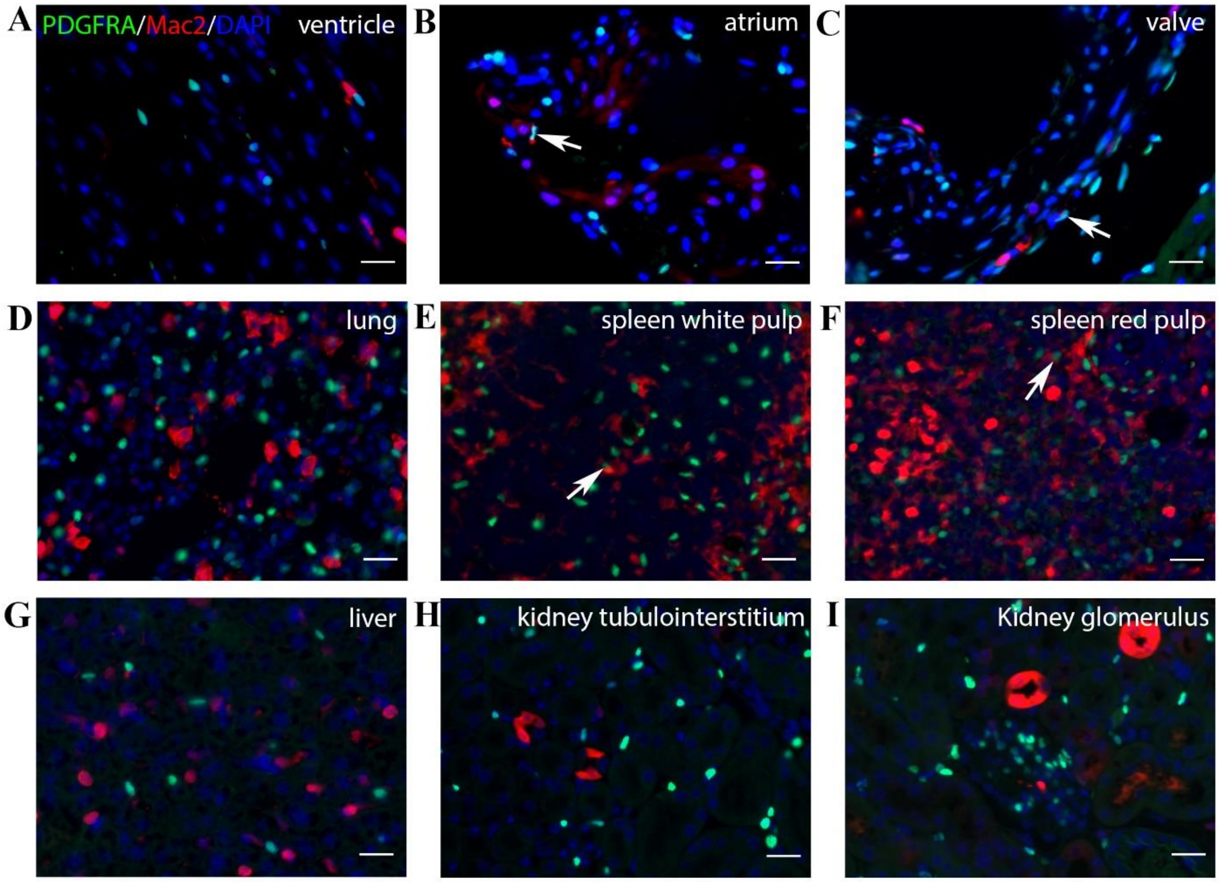

Supplemental figure V: Limited co-localization of PDGFRα and Mac2 in normal mouse organs. Heart, lung, spleen, liver and kidney were harvested from PDGFRα-EGFP reporter mice and processed for histological study. Dual immunofluorescent staining for GFP and Mac2 was used to identify PDGFRα+ fibroblasts that express Mac2. A small number of PDGFRα+Mac2+ cells were identified in the heart (A-C), lung (D), spleen (E, F), and liver (G) (arrows). Quantitative analysis is shown in Table 1. Scale bar: 20 μm.

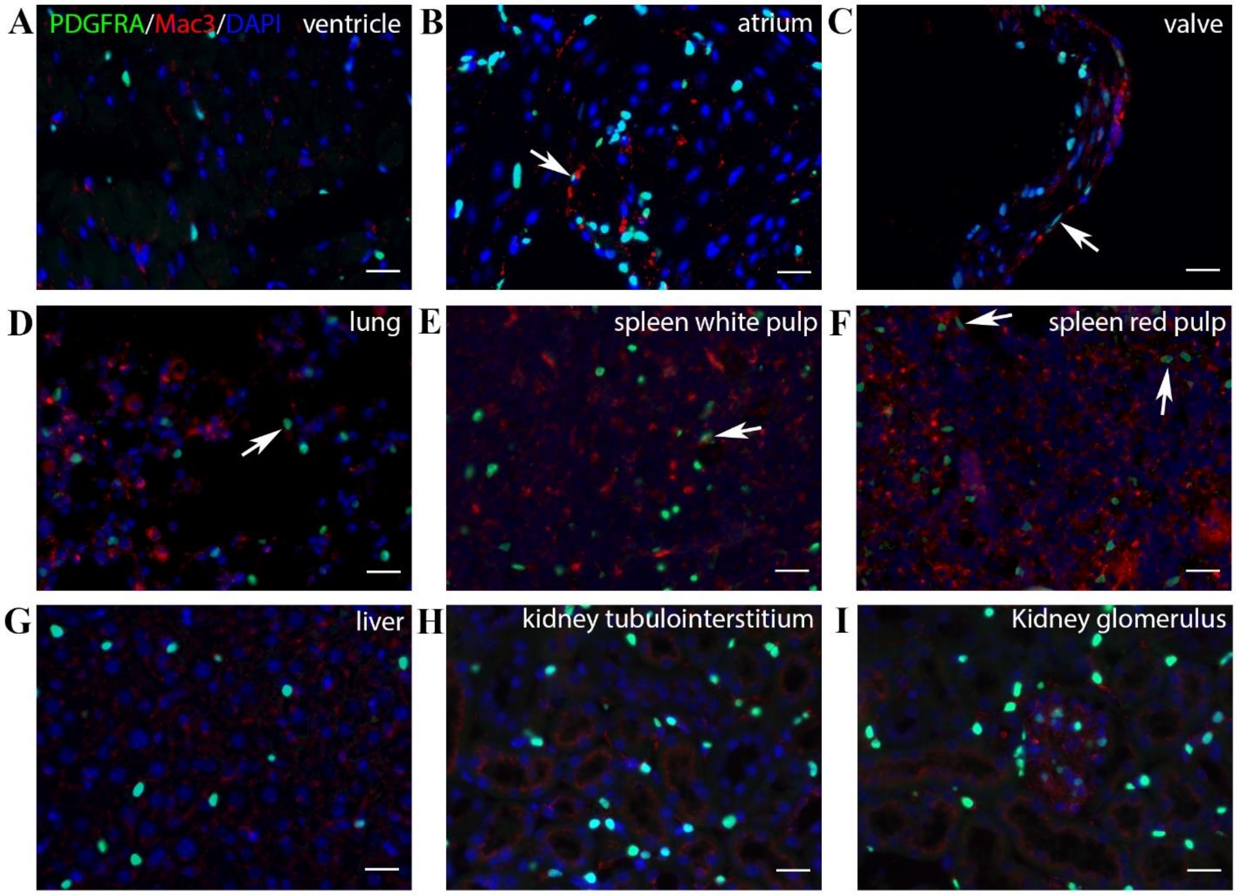

Supplemental figure VI: Limited co-localization of PDGFRα and Mac3 in normal mouse tissues. Heart, lung, spleen, liver and kidney were harvested from PDGFRα-EGFP reporter mice and processed for histology. Dual immunofluorescent staining for GFP and Mac3 was used to identify fibroblasts expressing Mac3 (arrows). A small number of PDGFRα+Mac3+ cells was identified in the heart (A-C), lung (D), spleen (E, F), kidney (H, I). Quantitative analysis is shown in Table 1. Scale bar: 20 μm.

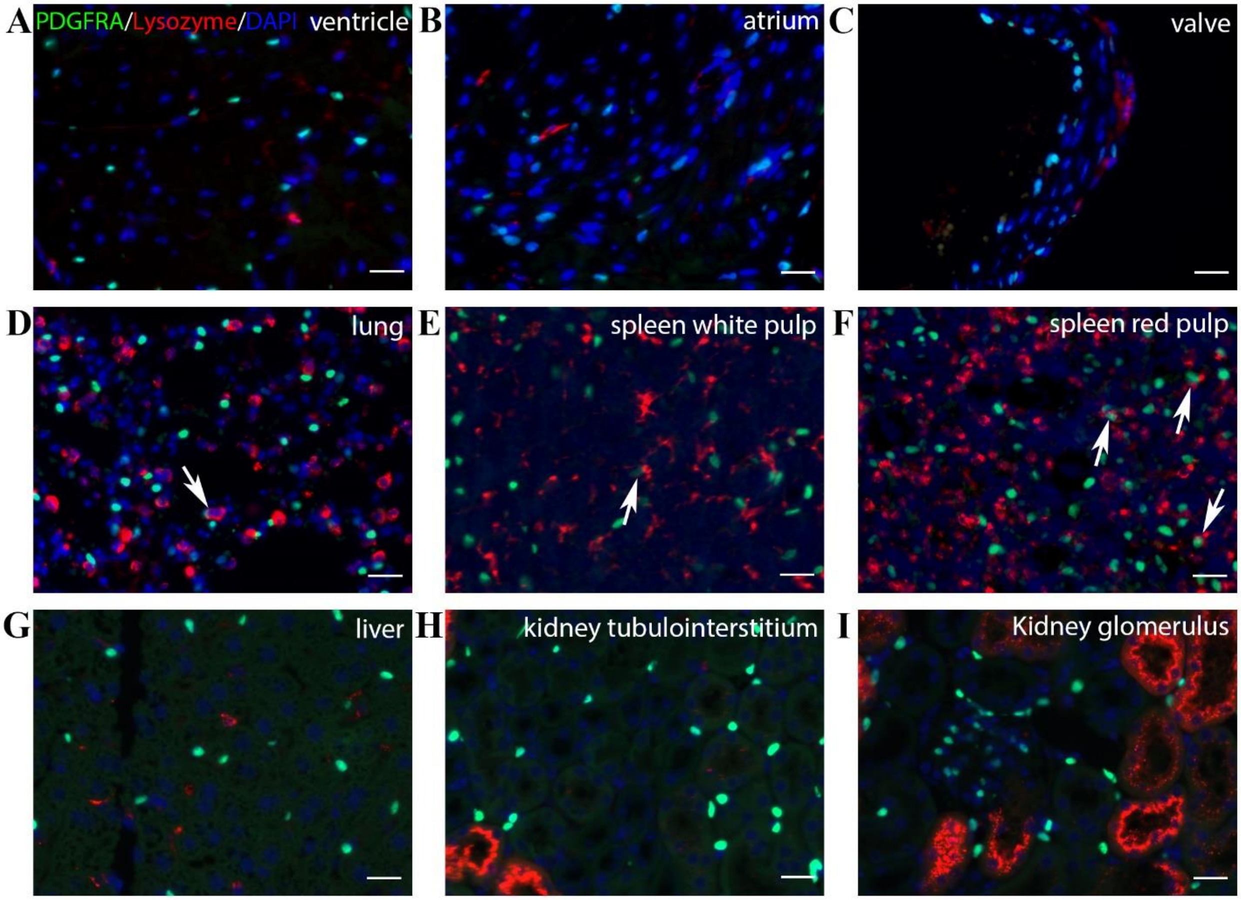

Supplemental figure VII: Limited co-localization of PDGFRα and LyzM in normal mouse tissues. Heart, lung, spleen, liver and kidney were harvested from PDGFRα-EGFP reporter mice and processed for histological studies. Dual immunofluorescent staining for GFP and Lysozyme (using anti-lysozyme antibody clone EPR2994(2)) was performed to identify PDGFRα+ fibroblasts that express LyzM. A small number of PDGFRα+/LyzM+ cells were identified in the heart (A-C), lung (D), spleen (E, F) (arrows), liver (G). Quantitative analysis is shown in Table 1. Scale bar: 20 μm.

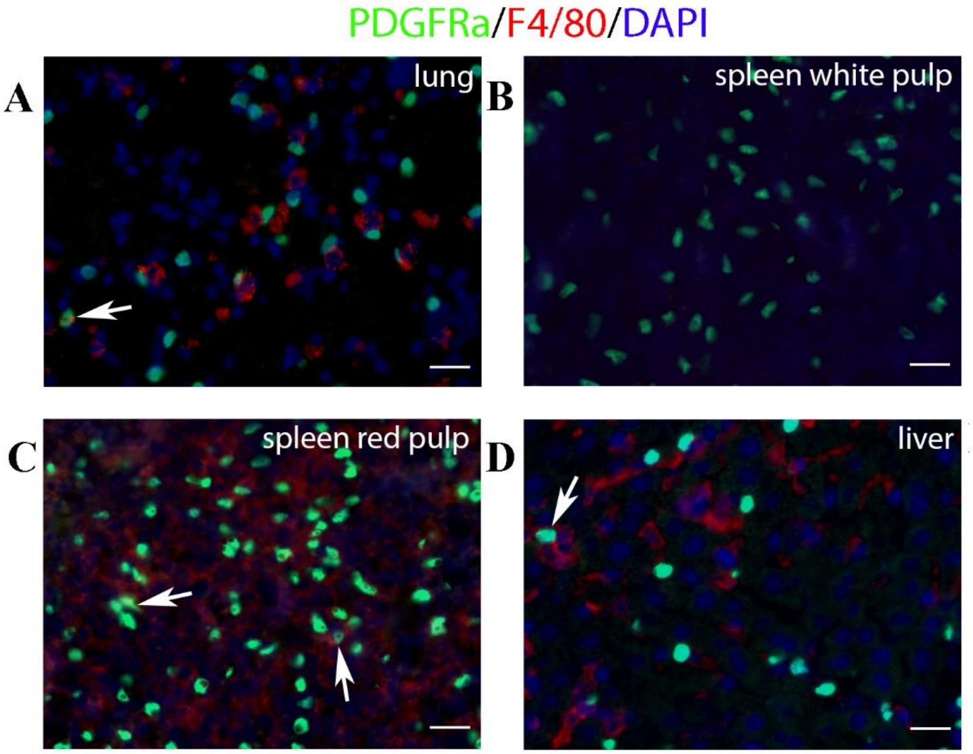

Supplemental figure VIII: Limited co-localization of PDGFRα and F4/80 in normal mouse organs. In order to examine whether F4/80 labels fibroblasts, tissues from fibroblast reporter PDGFRα-EGFP mice were stained with the anti-F4/80 antibody ab111101. Dual immunofluorescent staining for GFP and F4/80 was used to identify fibroblasts that were labeled for F4/80. A small number of PDGFRα+/F4/80+ cells was identified in the lung (A), spleen (B, C), and liver (D) (arrows). Quantitative analysis is shown in Table 1. Scale bar: 20 μm.

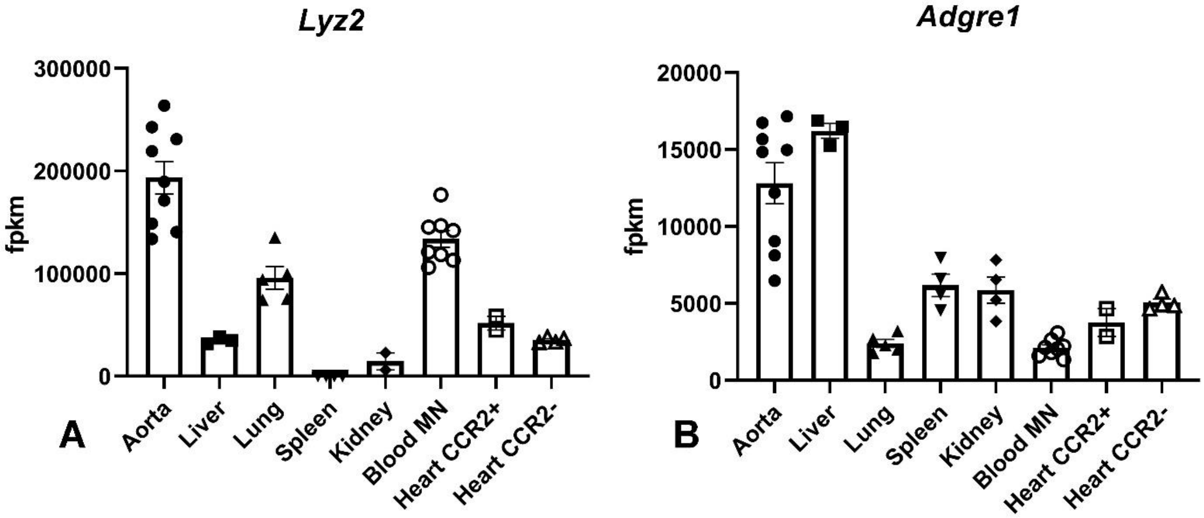

Supplemental Figure IX: Expression of *Lyz2* and *Adgre1*, the genes encoding lysozyme-M and F4/80 protein respectively in macrophages harvested from various organs. Data mined from the Immgen ULI Open Source Mononuclear Phagocytes Project (GEO accession number: GSE122108). A. Liver, spleen and kidney macrophages express lower levels of *Lyz2*, consistent with the reduced sensitivity of antibodies to lysozyme in detecting these cells. In contrast, *Lyz2* levels are high in lung macrophages and blood monocytes (MN). B. Cardiac macrophages express relatively low levels of *Adgre1*, in comparison to hepatic and spleen macrophages. Thus, reduced *Adgre1* expression may contribute to the absence of staining of cardiac macrophages with 3 different F4/80 antibodies (n=2-9/group).

SUPPLEMENTAL REFERENCES:

1 Cao, Q. *et al.* Renal F4/80+ CD11c+ mononuclear phagocytes display phenotypic and functional characteristics of macrophages in health and in adriamycin nephropathy. *J Am Soc Nephrol* **26**, 349-363, doi:10.1681/ASN.2013121336 (2015).

2 Hume, D. A. & Gordon, S. Mononuclear phagocyte system of the mouse defined by immunohistochemical localization of antigen F4/80. Identification of resident macrophages in renal medullary and cortical interstitium and the juxtaglomerular complex. *J Exp Med* **157**, 1704-1709, doi:10.1084/jem.157.5.1704 (1983).

3 Stamatiades, E. G. *et al.* Immune Monitoring of Trans-endothelial Transport by Kidney-Resident Macrophages. *Cell* **166**, 991-1003, doi:10.1016/j.cell.2016.06.058 (2016).

4 Hofmeister, A. *et al.* Development of a new macrophage-specific TRAP mouse (Mac(TRAP)) and definition of the renal macrophage translational signature. *Sci Rep* **10**, 7519, doi:10.1038/s41598-020-63514-6 (2020).

5 Salei, N. *et al.* Selective depletion of a CD64-expressing phagocyte subset mediates protection against toxic kidney injury and failure. *Proc Natl Acad Sci U S A* **118**, doi:10.1073/pnas.2022311118 (2021).

6 Lever, J. M. *et al.* Resident macrophages reprogram toward a developmental state after acute kidney injury. *JCI Insight* **4**, doi:10.1172/jci.insight.125503 (2019).

7 Ide, S. *et al.* Yolk-sac-derived macrophages progressively expand in the mouse kidney with age. *Elife* **9**, doi:10.7554/eLife.51756 (2020).

8 Inoue, T., Plieth, D., Venkov, C. D., Xu, C. & Neilson, E. G. Antibodies against macrophages that overlap in specificity with fibroblasts. *Kidney Int* **67**, 2488-2493, doi:10.1111/j.1523-1755.2005.00358.x (2005).

9 Gersch, C. *et al.* Mast cells and macrophages in normal C57/BL/6 mice. *Histochem Cell Biol* **118**, 41-49 (2002).

10 Heidt, T. *et al.* Differential contribution of monocytes to heart macrophages in steady-state and after myocardial infarction. *Circ Res* **115**, 284-295, doi:10.1161/CIRCRESAHA.115.303567 (2014).

11 Aurora, A. B. *et al.* Macrophages are required for neonatal heart regeneration. *J Clin Invest* **124**, 1382-1392, doi:10.1172/JCI72181 (2014).

12 Lorchner, H. *et al.* Myocardial healing requires Reg3beta-dependent accumulation of macrophages in the ischemic heart. *Nat Med* **21**, 353-362, doi:10.1038/nm.3816 (2015).

13 Iqbal, A. J. *et al.* Human CD68 promoter GFP transgenic mice allow analysis of monocyte to macrophage differentiation in vivo. *Blood* **124**, e33-44, doi:10.1182/blood-2014-04-568691 (2014).

14 Kingery, J. R. *et al.* Leukocyte iNOS is required for inflammation and pathological remodeling in ischemic heart failure. *Basic Res Cardiol* **112**, 19, doi:10.1007/s00395-017-0609-2 (2017).

15 Shigeta, A. *et al.* Endocardially Derived Macrophages Are Essential for Valvular Remodeling. *Dev Cell* **48**, 617-630 e613, doi:10.1016/j.devcel.2019.01.021 (2019).

16 Bu, J. *et al.* The GABAA Receptor Influences Pressure Overload-Induced Heart Failure by Modulating Macrophages in Mice. *Front Immunol* **12**, 670153, doi:10.3389/fimmu.2021.670153 (2021).

17 Li, J. *et al.* CD226 deletion improves post-infarction healing via modulating macrophage polarization in mice. *Theranostics* **10**, 2422-2435, doi:10.7150/thno.37106 (2020).

18 Dewald, O. *et al.* CCL2/Monocyte Chemoattractant Protein-1 regulates inflammatory responses critical to healing myocardial infarcts. *Circ Res* **96**, 881-889 (2005).

19 Dewald, O. *et al.* Of mice and dogs: species-specific differences in the inflammatory response following myocardial infarction. *Am J Pathol* **164**, 665-677 (2004).
